# Timing of the Saalian- and Elsterian glacial cycles and the implications for Middle – Pleistocene hominin presence in central Europe

**DOI:** 10.1038/s41598-018-23541-w

**Published:** 2018-03-23

**Authors:** Tobias Lauer, Marcel Weiss

**Affiliations:** Max-Planck-Institute for Evolutionary Anthropology, Department of Human Evolution; Deutscher Platz 6, D-04103 Leipzig, Germany

## Abstract

By establishing a luminescence-based chronology for fluvial deposits preserved between the Elsterian- and Saalian tills in central Germany, we obtained information on the timing of both the Middle Pleistocene glacial cycles and early human appearance in central Europe. The luminescence ages illustrate different climatic driven fluvial aggradation periods during the Saalian glacial cycle spanning from 400–150 ka. The ages of sediments directly overlying the Elsterian till are approximately 400 ka and prove that the first extensive Fennoscandian ice sheet extension during the Quaternary correlates with MIS 12 and not with MIS 10. Furthermore, the 400 ka old fluvial units contain Lower Paleolithic stone artefacts that document the first human appearance in the region. In addition, we demonstrate that early MIS 8 is a potential date for the onset of the Middle Paleolithic in central Germany, as Middle Paleolithic stone artefacts are correlated with fluvial units deposited between 300 ka and 200 ka. However, the bulk of Middle Paleolithic sites date to MIS 7 in the region. The fluvial units preserved directly under the till of the southernmost Saalian ice yield an age of about 150 ka, and enable a correlation of the Drenthe stage to late MIS 6.

## Introduction

The timing of the Middle Pleistocene glacial-interglacial cycles and the feedback mechanisms between climatic shifts and earth-surface processes are still poorly understood. This is largely due to a lack of dating results of sediments representing the advance and retreat of Middle Pleistocene ice sheets, as well as periglacial, interglacial or interstadial environments.

One key issue is the onset of the Elsterian glaciation, which was the first and southern-most ice advance of Fennoscandian ice sheets into the European continent^1^, and its relationship with the Middle Pleistocene Revolution (hereafter MPR). The MPR describes the shift from 41 ka–100 ka climate cycles that occurred ~1.5 Ma–0.5 Ma ago^2^. That significant change in climate cyclicity occurred without any change in external isolation forcing^3^, and huge ice sheets might have played a key role in this^4,5^, as they balanced insolation maxima^3^. To understand the feedback between earth ice sheets and the MPR, it is crucial to understand when this southern-most advance of Fennoscandian glaciers occurred. The timing of this important event is still a matter of debate^1,6–9^.

Several Early- and Middle-Pleistocene expansions of the British and Scandinavian ice are documented from the North Sea Basin^8–12^. The first basin-wide ice sheet expansion corresponds with Marine Isotope Stage (hereafter MIS) 12^8,13^, but it is debatable if this ice advance correlates to what is, per definition, the Elsterian glaciation because the typo region for the latter is located much further south in central Germany^14–17^ and resilient chronological data from this isn’t available yet. Consequently, the correlation between the Elsterian glaciation and MIS 10 is still discussed^6^. The latter is mainly linked to the stratigraphical position of the Elsterian prior to the Holsteinian interglacial^1^. For the Holsteinian, a correlation with MIS 11 or MIS 9 is still debated, as it is mainly linked to the chronology of the Bossel-profile^7,18^, Lower Saxony/Germany. The latter is regarded as a typo-profile for the Holsteinian and the corresponding U-series ages point to a correlation with MIS 9. Consequently, the German Stratigraphic Table^6^, which is a fundamental stratigraphical reference, states an age of 320-300 ka for the Holsteinian. However, potential problems with U/Th-dating of peat due to the open system behavior of U-series were recently outlined by Sierralta *et al*.^19^ for another Holsteinian type profile from Wedel, Schleswig-Holstein/Germany and the Paleolithic site of Schöningen, Lower Saxony/Germany. Furthermore, other studies^20–25^ deliver evidence of a correlation of the Holsteinain with MIS 11 and further clarification is needed.

With regard to the Saalian glacial cycle, more chronological data is needed to reconstruct the timing and extension of the two significant ice advances and spatial variations among different parts of Europe.

Furthermore, during most of the Saalian glacial cycle central Europe was characterized by periglacial environmental conditions^14,17,26^. Chronological data of sediment archives representing periglacial but also potentially warmer Saalian climate periods are very sparse until now. Therefore, there is a lack of knowledge about the response of sedimentary systems to climatic shifts during the Saalian period.

A better understanding of Middle Pleistocene palaeoenvironmental changes is also of high relevance for the understanding of human dispersal into central Europe, as well as the timing of technological changes within the archaeological record. The Middle Pleistocene is the time period from which we start to have major archaeological evidence for human presence together with the emergence of the Middle Paleolithic (‘MP’; see e.g^27–31^.) in central Europe. Therefore, the oldest traces of human appearance in central Germany post-date the Elsterian glaciation (see e.g^27,28,32–38^). Whether this first glaciation eroded traces of prior human occupation, or whether the Baltic flint transported to large parts of the central European lowlands attracted humans as a high quality raw material source, remains an open question. Nevertheless, the age of the Elsterian glaciation is crucial for our understanding of when humans (re-)populated the European lowlands. Additionally, a higher resolution dating of the time period between the Elsterian and Saalian glaciations may help to understand when and under which palaeoenvironmental conditions the transition from the Lower to the Middle Paleolithic occurred in the study area.

**Figure 1:**
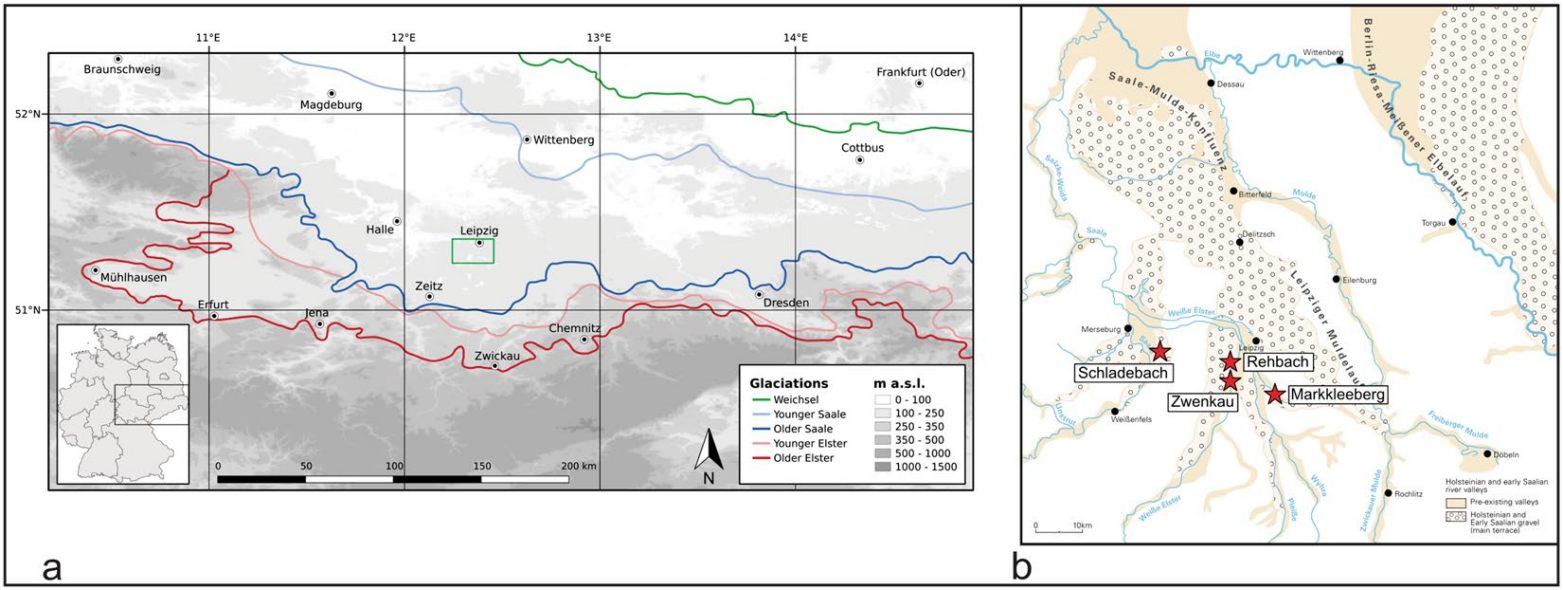
**(a)** Ice marginal positions of the main ice advances into central Germany^17^ and **(b)** map showing the distribution of the Saalian Main Terrace (SMT) in the study area and the location of investigated sites. (**a**) The green box marks the study area. The 2 ice advances (Older and Younger Elster) during the Elsterian stage are also termed Zwickau phase (1^st^ ice advance) and Markranstädt Phase (2^nd^ ice advance) in the Leipzig Lowlands. The tills are separated by melt- water-sands of the so called “Miltitz- Intervall”. Both significant ice advances during the Elsterian stage are estimated to have occurred during the late Elsterian^17,96,97^. During the Saalian glacial cycle, the first and southern-most ice sheet extension into the Leipzig lowlands (Older Saale) reached the city of Zeitz (Zeitz-phase). The Zeitz-phase correlates to the late Saalian Drenthe stadial^1,17^. The Younger Saalian ice advance (Warthe) did not reach the study area. Warm stage deposits between the different Saalian ice advances are not documented^1^. We composed the map using SRTM 1Arc-Second Global data for elevation (https://earthexplorer.usgs.gov/) and processed it with the GNU General Public License software QGIS Version 2.18 (http://www.qgis.org/en/site/). **(b)** The SMT was formed by several rivers within the Saale-Elbe area and the term “Saalian” refers to the formation of the fluvial units during the Saalian glacial cycle. The terminus Main “Terrace”^98^ is not used in a morphological sense. The stacked fluvial units accumulated in several phases between the Holsteinian interglacial and the Saalian ice advance of the Zeitz-phase^17,39,52^. The map was redrawn from Eissmann^99^ using Adobe Illustrator CS6.

The most suitable area to investigate the above questions is the type area for the Elsterian- and Saalian glacial cycles, located in central Germany^15^ (Saxony, Saxony-Anhalt and Thuringia, Fig. 1a). This is where the gravel deposits of the rivers Saale- and Elster intertwine with tills and meltwater deposits of both glacial cycles in proximity to the maximum extensions of the Fennoscandian ice sheets in Central Europe^17,39–42^. To obtain chronological data for the timing of the Elsterian- and Saalian glacial cycles, we used feldspar luminescence dating stimulating the feldspar-luminescence signal at elevated temperatures^43–46^^.^

As tills cannot be dated using luminescence dating, the fluvial units of the so called ‘Hauptterrasse’ (Fig. 1b) or Saalian Main Terrace (hereafter SMT) are pertinent to these issues. Its stacked sediment-units are preserved between the tills of the Elsterian and Saalian glaciations.

Additionally, the basal part of the SMT is associated with Middle- and Lower Paleolithic stone artifacts^32,33,47–51^.

By dating the SMT, it is therefore possible:

A. to get reliable chronological control for the Elsterian and Saalian ice advances.
B. to reconstruct the climatically driven fluvial aggradation within the Saalian glacial cycle.
C. to obtain minimum ages for the embedded Middle- and Lower Paleolithic stone artefacts.

## Results

### Stratigraphy

The lithology and facies architecture of the SMT as well as the stratigraphical position of associated paleolithic artefact findings have been well documented in several studies^14,16,17,32,39,42,50,52,53^. The very bottom part of the SMT, when preserved, is built by the Corbicula-fluvial unit which contains warm-stage fossils^54,55^. This points to an initial formation of the SMT under warm climate conditions, but a reworking of the fossils must be considered. All overlying units of the SMT have been attributed to cold climate^56^ as evidenced by the fluvial facies architecture and cryoturbation features including several levels of ice wedges. Furthermore, mammal remains such as *Mammuthus primigenius* or *Ovibos moschatus* are suggestive of the formation of the SMT in a periglacial environment.

Only a small number of infrared-radiofluorescence ages of SMT- sediments, ranging from 306 ± 23 ka to 227 ± 15 ka^57^ are available from different sites in eastern Germany, however these ages were presented without stratigraphical context, making the interpretation challenging.

The currently exposed lithostratigraphy of the SMT sites of Rehbach, Zwenkau and Schladebach/Wallendorf was documented during recent luminescence sampling-campaigns. The site of Markkleeberg is no longer accessible and luminescence samples were provided by the Freiberg (Saxony) dating laboratory. The lithological description of the site is based on the work of E. Miersch^58^.

**Figure 2:**
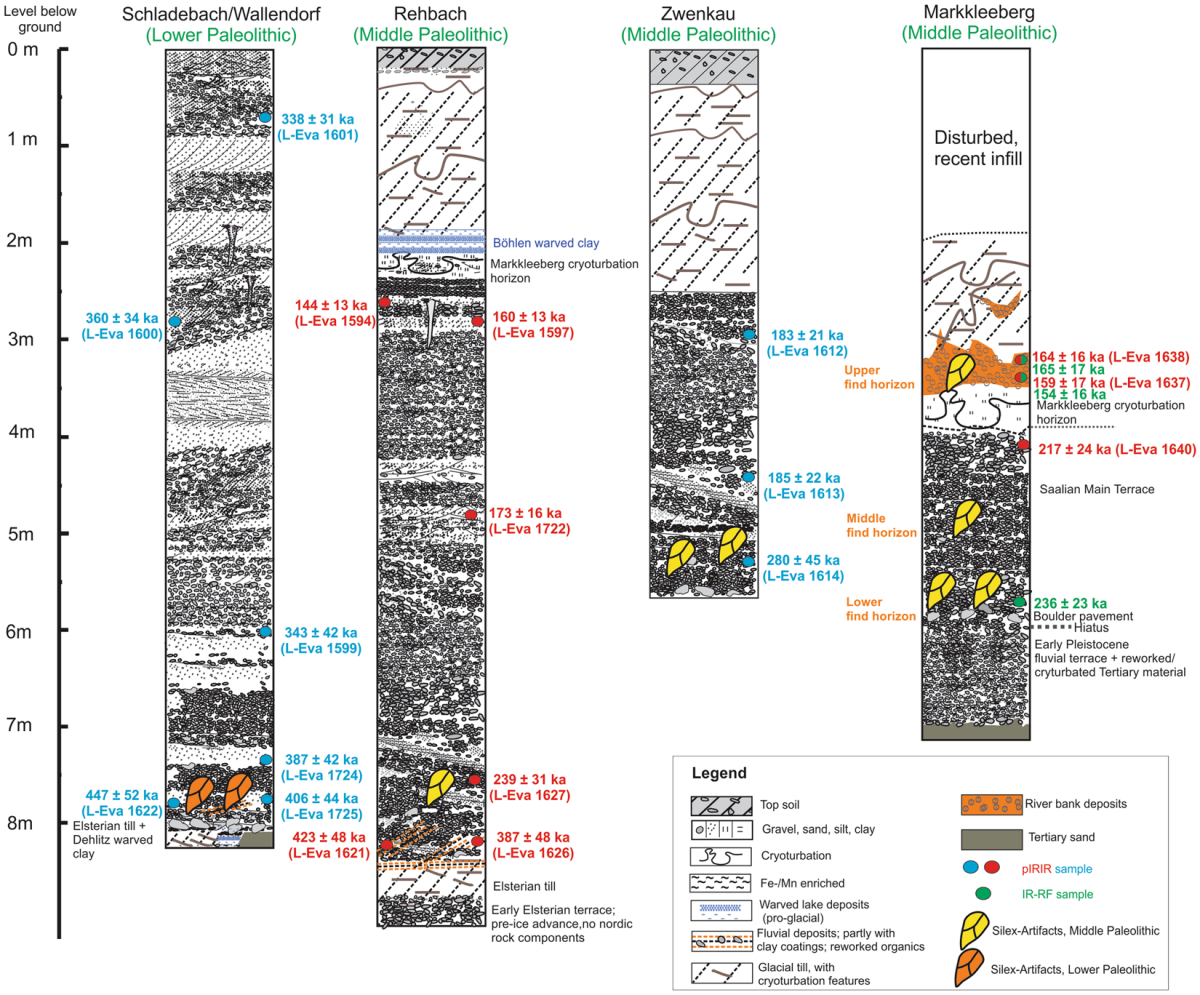
Lithostratigraphy of the fluvial units exposed at Schladebach/Wallendorf, Rehbach, Zwenkau and Markkleeberg including pIRIR_290_ luminescence ages. A summary of all luminescence ages is given in Supplementary Table S2. The green-colored ages are infrared-radiofluorescence ages formerly presented by Krbetschek *et al*.^61^ (see results chapter). At Schladebach and Rehbach, the Saalian Main Terrace is visibly capping the Elsterian till. Only the first Elsterian till is preserved whereas the upper Elsterian till was eroded and only a boulder pavement as a till-residuum is found. At Markkleeberg, only the boulder pavement is preserved. At Schladebach and Rehbach, fragments of the ice-dammed lake sediments of the Dehlitz-Leipzig warved clay^41^ are preserved below the SMT. The SMT itself is mostly horizontally bedded but also shows some cross-beddings. The upper fluvial sand and gravel are characterized by permafrost features such as ice wedges, and at Rehbach and Markkleeberg, the “Markkleeberg cryoturbation horizon” is preserved. The Markkleeberg cryoturbation horizon is a wide-spread, silt-rich unit that was not found at the outcrop at Zwenkau. The SMT is concordantly capped by the Böhlen warved clays and the up to 2 m thick Saalian till (Drenthe). The fluvial sequence in Markkleeberg was formed by the Pleiße/Gösel-river system. The gravel of the SMT at Rehbach and Zwenkau were deposited by the Weiße Elster river and the petrographic composition shows a high percentage of Nordic material (e.g. flint) as well as quartz, greywacke and chert (Kieselschiefer) documenting the catchment area of the Weiße Elster river in eastern Thuringia and the Vogtland. The SMT deposits at Schladebach correlate to the Saale-Unstrut river system^14^. The gravel composition of these deposits is characterized by high percentages of limestone originating from the Triassic sediment formations located south of the section in addition to Scandinavian rock components including flint.

The sedimentary units of all sites under investigation are outlined in Fig. 2, which also illustrates the stratigraphical position of stone artefacts.

### Luminescence chronology

#### Schladebach

The luminescence samples taken from the very bottom part of the SMT exposed at Schladebach, which contain reworked clay and organic rich sediments and directly cap the Elsterian till, yield ages ranging from 387 ± 42 ka –447 ± 52 ka (Fig. 2; Supplementary Table S2). The ages overlap within the error-range and point to an initial period of fluvial aggradation of the SMT during MIS 11 or early MIS 10^59^. The decalcification of the basal gravels in the Schladebach/Wallendorf SMT and the presence of mollusks^60^ support an onset of the fluvial aggradation during moderate climate conditions^33^. These luminescence ages deliver a minimum age for the embedded Lower Paleolithic stone artefacts of approximately 400 ka.

The pIRIR_290_ luminescence age estimates obtained from the upper- and middle part of the Schladebach section are 343 ± 42 ka, 360 ± 34 ka and 338 ± 31 ka. This age-cluster points to the aggradation of several meters of sand and gravel during MIS 10.

#### Rehbach

The luminescence ages of Rehbach are indicative of 4 phases of fluvial aggradation. The ages obtained from the very bottom part of the SMT, preserved fragmentarily above the Elsterian till, are 423 ± 48 and 387 ± 48 ka, and are similar to the corresponding luminescence ages obtained at Schladebach. Therefore, at Rehbach the initial formation of the SMT correlates to MIS 11/ early MIS 10 too.

The capping fluvial unit is dated to 239 ± 31 ka and correlates to late MIS 8 or MIS 7. Therefore, a significant chronological gap of around 150 ka is documented within the Rehbach sedimentary sequence.

The central part of the SMT at Rehbach is dated to 173 ± 16 ka, suggesting a 3^rd^ period of fluvial aggradation during MIS 6.

The top part of the SMT, which directly underlies the till of the southern-most ice sheet extension (Zeitz phase) during the Saalian glacial cycle, yields luminescence ages of 144 ± 13 ka and 160 ± 13 ka. These ages correlate to a later period of MIS 6, and constitute maximum ages for the till of the Zeitz-phase (Drenthe).

#### Zwenkau

At Zwenkau, only approximately 3 meters of fluvial sand and gravel are exposed. The bottom portion was dated to 280 ± 45 ka (MIS 8). The capping unit, here discordantly underlying the Saalian till of the Zeitz phase, is dated to 185 ± 22 and 183 ± 21 ka. This suggests that the main part of the preserved SMT at Zwenkau dates to early MIS 6 and can chronostratigraphically be correlated to the middle part of the Rehbach-section. Late MIS 6 fluvial sediments are not preserved at Zwenkau.

#### Markkleeberg

The archeologically important site of Markkleeberg is no longer accessible due to the closing of the former pit. The dating material from the SMT was taken by Matthias Krbetschek (1956–2012) from the University of Freiberg (Saxony) in the early 2000s. Some of the K-feldspar samples were kindly provided to us by Freiberg University. Unfortunately, no material was available from the bottom portion of the SMT, from which most of the stone artefacts derive. The only chronological information of this basal unit is based on preliminary infrared-radiofluorescence (IR-RF) ages^42,57,61^ pointing to an aggradation around 250 ka (236 ± 23 ka)^61^. However, a quality-validation of this IR-RF age is not possible.

The pIRIR_290_ luminescence ages from the top-part of the SMT at Markkleeberg are 217 ± 24 ka (the sediments underlying the Markkleeberg silt and cryoturbation horizon), and the unit concordantly underlying the Saalian till yields luminescence ages of 164 ± 16 and 159 ± 17 ka. The pIRIR_290_ ages are in very good agreement with the IR-RF ages obtained from the same samples presented by Krbetschek *et al*.^61^.

### Archaeology

**Figure 3:**
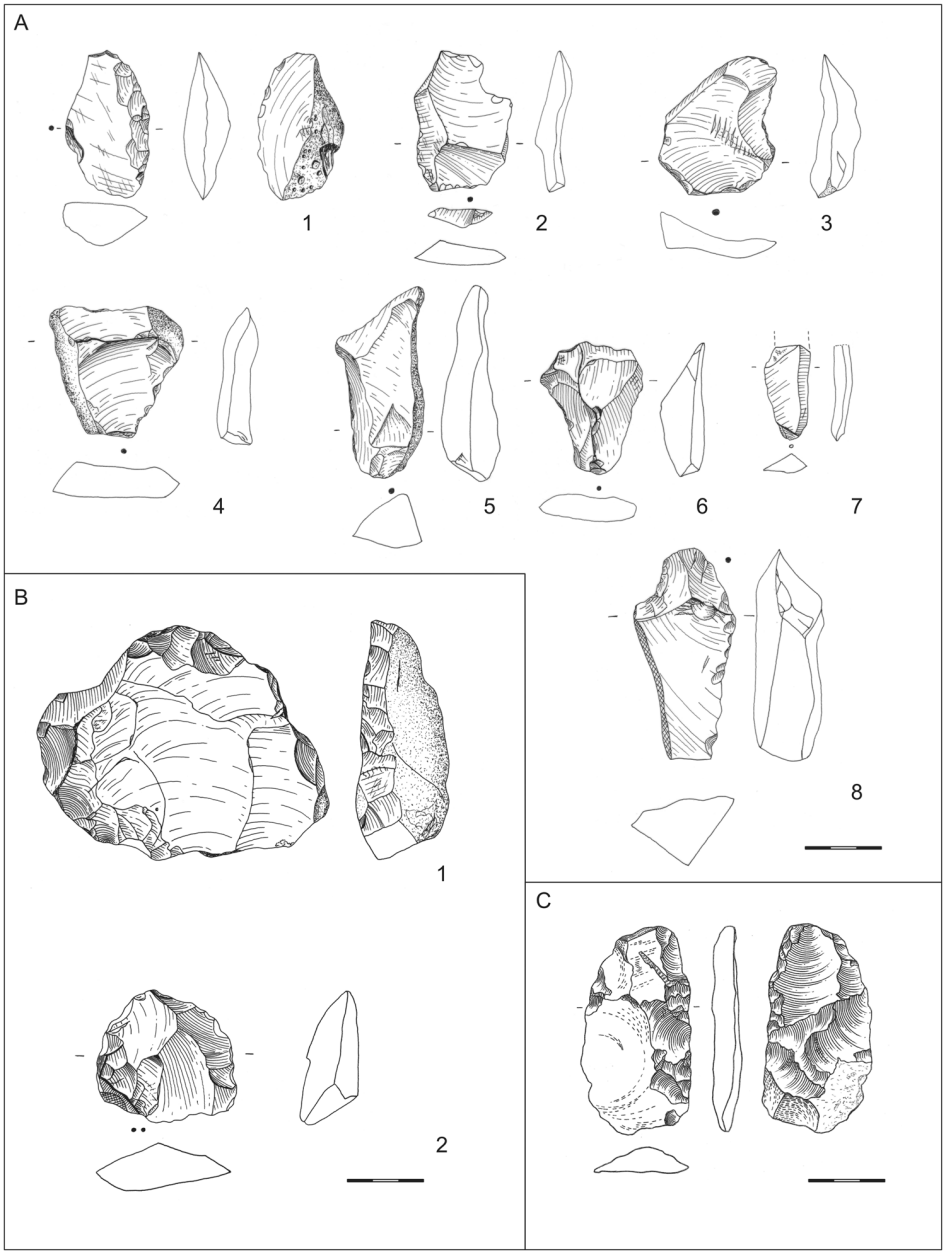
Artefacts found during sampling for luminescence dating and additional surveys in Schladebach (**A**), Rehbach (**B**) and during a formerly survey in Zwenkau-“Bösdorf” (**C**). A: 1–scraper (11263:1000:8); 2–7–flakes (2–11263:1000:1; 3–11263:1000:23; 4–11263:1000:11; 5–11263:1000:13; 6–11263:1000:24; 7–11263:1000:3); 8–flake with potentially retouched notch (11263:1000:29); B: 1–prepared core (REH-09/1/2); 2–flake (REH-09/1/3); C: bifacial scraper (BQF-04/1/1). Scale: ½ original size. Numbers given for Schladebach are the inventory numbers of the Landesamt für Denklmalpflege und Archäologie Sachsen-Anhalt–Landesmuseum für Vorgeschichte; the numbers given for Rehbach and Bösdorf are the inventory numbers of the Landesamt für Archäologie Sachsen. Drawings: A and B: M. Weiss; C: W. Bernhardt.

**Figure 4:**
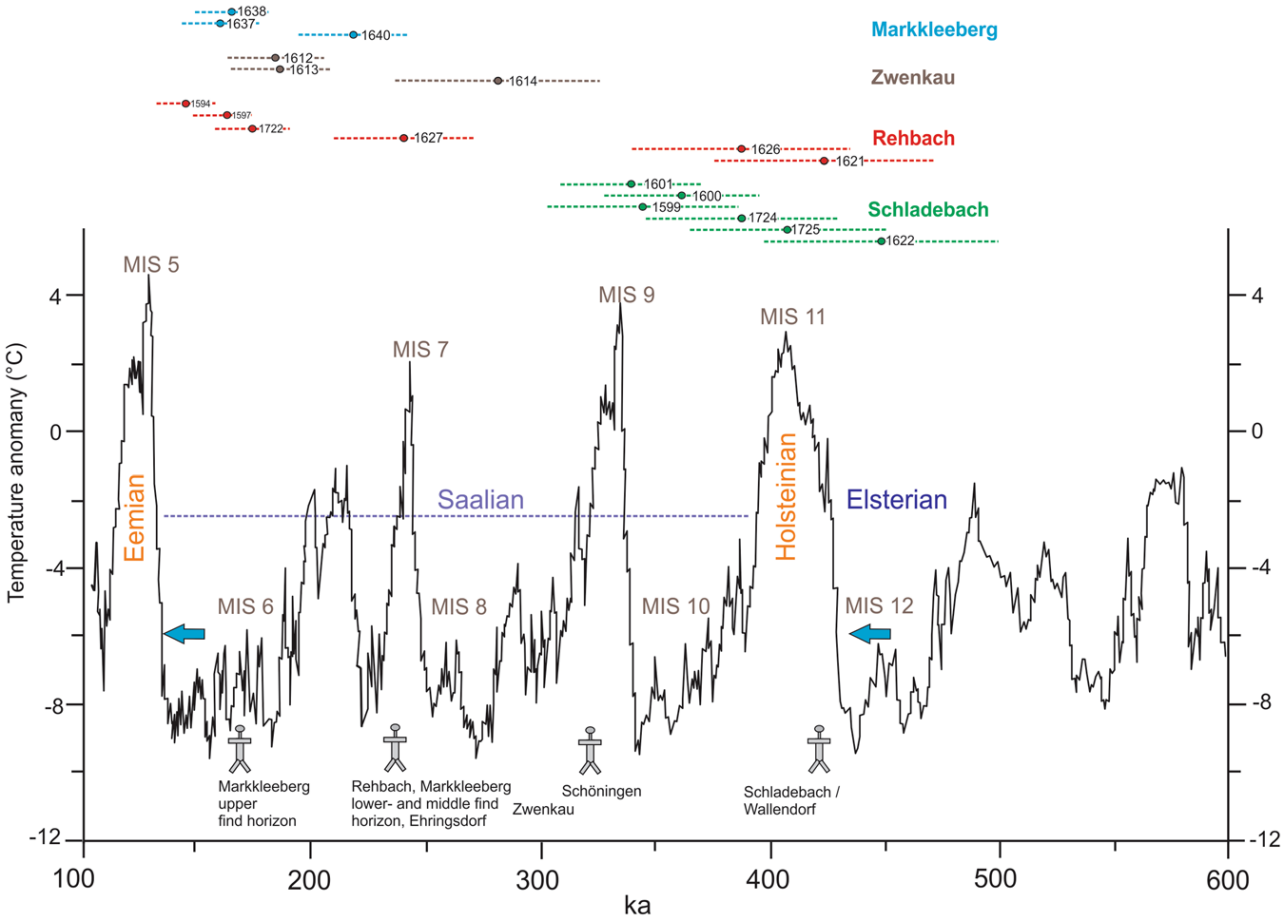
Middle Pleistocene climatic fluctuations^100^ as well as luminescence ages from this study indicating periods of Middle-Pleistocene fluvial aggradation of the SMT. Within the bottom part of the graph, next to the boxes with site labels, the direct dated sites Weimar-Ehringsdorf^71,72,82^ and Schöningen^34,78^ are added to complete the picture of human presence in central Germany. The MIS boundaries^59^ referred to in that paper are: 13/12 = 478 ka; 12/11 = 424 ka; 11/10 = 374 ka; 10/9 = 337 ka; 9/8 = 300 ka; 8/7 = 243 ka; 7/6 = 191 ka; 6/5 = 130 ka. First traces of humans in central Germany (Schladebach/Wallendorf) are most likely connected to the Holsteinain (MIS 11). The blue arrows mark a: the first significant ice advances of Fennoscandian glaciers during the Quaternary (Elsterian glacial cycle) within MIS 12 and b: the southernmost ice sheet extension during the Saalian glacial cycle (Zeitz-phase; Drenthe).

From the middle of the 20^th^ century until today, more than 6700 Lower Paleolithic artefacts were recovered from the base of the SMT sequence and the coarse gravel dump at the gravel pits of Schladebach and Wallendorf^32,33^. During luminescence sampling and additional surveys, a number of additional finds were documented including 6 fragmented animal bones, 10 simple flake cores (Supplementary Figures S23–S25), 16 flakes and 3 tools (Fig. 3A), all of which derive from the exposed basal parts of the SMT sequence (see Supplementary Figures S19–S21). The artefacts are primarily of Lower Paleolithic character, though only one flake has a slightly faceted platform (see Supplementary Tables S3 and S4), indicating a trend towards the Middle Paleolithic. This slight trend has already been observed in the earlier collections^32,33^. The luminescence ages of approximately 400 ka (Fig. 2) indicate a human presence in central Germany that is characterized by a Lower Paleolithic stone tool industry including sparse MP features^32,33^ during MIS 11 or early MIS 10 (Fig. 4). No knapping sites were documented in the basal gravels, either within our own or previous surveys. Instead, the artefacts are scattered along the whole basal SMT sequence. Sharp edged artefacts are rare in the older collections^33^. During our own survey, the recovered artefacts (see Supplementary Tables S3 and S4) are a mixture of lightly edge damaged pieces (e.g. Fig. 3A-7), edge damaged flakes (e.g. Fig. 3A-2) and rolled artefacts (e.g. Fig. 3A-5). Indications show that artefacts were predominantly reworked during the onset of the fluvial aggradation. Therefore, they are likely slightly older than the ages of the SMT at the Schladebach pit presented here. In other words, the ages for the sediment that contains the artefacts should be interpreted as a minimum age for the human presence at the site. As the basal sediments of the SMT suggest temperate climatic conditions^60^, and the artefacts are found in sediments overlying Elsterian glacial deposits, an MIS 11 age for the artefacts can be inferred. Given that fact that the artefacts only occur within the basal layers of the sequence as well as the ages for the whole sequence are older than MIS 9 (except the age on top of the gravel accumulation; Fig. 2), an age younger than MIS 10 for the artefacts along with the human presence can be excluded.

In conclusion, Schladebach/Wallendorf is now the oldest directly dated archaeological site in central Germany^27^ and is among the oldest sites in central Europe (the Biśnik cave in Poland provided some similar and some older dates, predating MIS 12^62^).

From the Middle Paleolithic site of Markkleeberg, we were able to date two of the three reported find layers^49^ present in the SMT sequence (Fig. 2): The upper find horizon, where artefacts were potentially preserved in primary position, yields an age of about 160 ka. This is evidence for human presence in central Germany during cold conditions prior to the first Saalian ice advance. The upper part of the Middle find horizon, where rolled artefacts in secondary context were excavated^49^, has an age of approximately 220 ka. Additionally, the IR-RF age (236 ± 23 ka) from the bottom part of the sequence (Krbetschek *et al*.^61^) indicates an MIS 7 age of these artefacts.

The MP artefacts from the former open-cast brown coal mine of Zwenkau were recovered from the base of the exposed SMT sequence. The already published assemblages^50^ were named after the former villages within the Zwenkau quarry “Eythra” and “Bösdorf”, which were destroyed by mining. The date for the base of the SMT presented here (Fig. 2) provides an age of 280 ± 45 ka for the artefacts, and is characterized by a sophisticated MP bifacial tool production system^50^. In the area of the western slope, from which the luminescence samples derive, a bifacial scraper was found in stratigraphic position at the basal part of the SMT during former surveys (Fig. 3C, pers. comm. W. Bernhardt, November 20^th^ 2017, who recovered and documented the artefact). The luminescence ages demonstrate the presence of humans with a fully developed MP stone tool industry between early MIS 8 and MIS 7 in central Germany (Fig. 4).

During sampling for luminescence dating in the gravel pit of Rehbach, a typical MP Levallois core and a flake were found (Fig. 3B) within dislocated SMT basal gravels. Additionally, a damaged, potentially artificial flint flake was found in stratigraphic position within the approximately 240 ka old gravel unit (see Supplementary Figure S27), that makes a correlation of all artefacts to that unit likely.

The age of around 240 ka for the basal fluvial sediments at Rehbach is younger compared to the age of around 280 ka obtained at the basal sedimentary sequence at Zwenkau, especially if the sites are part of the same fluvial terrace. Nevertheless, the age for the find bearing basal part of the SMT at Rehbach of 239 ± 31 ka overlaps the lower error range with the age obtained for the basal part of the SMT at Zwenkau (Fig. 2; Fig. 4). This overlap confirms the MP human presence in central Germany around MIS 8–7.

## Discussion and Conclusion

### Implications of the SMT chronology for the timing of the Fennoscandian ice sheet extension, and the age of the Holsteinian

The ages of the lower-most gravel unit of the SMT, ranging from 447 ± 52 ka-387 ± 48 ka, post-date the Elsterian till. This highlights the fact that the Elsterian glacial cycle cannot be correlated with MIS 10 and therefore correlates to MIS 12. These age estimates deliver the first resilient chronological control for the Elsterian glacial cycle from its typo region and demonstrate that there is limited temporal gap between the end of Middle Pleistocene Revolution and the onset of the huge Middle Pleistocene glaciations in Europe.

As the Elsterian glacial cycle is terminated by the Holsteinian interglacial, the ages presented here support the suggestion of a correlation of the Holsteinian with MIS 11.

The top part of the SMT yields luminescence ages ranging from 164 ± 16 ka - 144 ± 13 ka, and was deposited during the later MIS 6. The stratigraphic position of theses layers directly under the Saalian till or Saalian warved clays shows that the southernmost Saalian ice advance in the region occurred during late MIS 6, hence at the end of the Saalian glacial cycle. The new data delivers important supplementary ages to the recently by Lang *et al*.^63^ published chronological data for the extension of the Saalian ice sheet into Germany. The pIRIR_290_ luminescence ages demonstrate that there might have been spatial- and chronological variations for the Middle-Pleistocene Fennoscandian ice sheet oscillations. For the region of northern Germany Roskosch *et al*.^64^ documented a higher number of Middle Pleistocene ice advances relative to our study area. More resilient chronological data of sediments correlating to ice sheet fluctuations along a European transect may be mandatory to better understand these spatial differences in the future.

### Driving forces and timing of fluvial aggradation within the SMT

The luminescence age estimates of the SMT point to several periods of fluvial aggradation spanning from about 400 ka–150 ka. The exposed fluvial sand and gravel cover a time span of approximately 250 ka, and the accumulation of the sedimentary sequences might be explained by the interplay of relatively rapid fluvial aggradation interrupted by periods of erosion or less fluvial activity.

### MIS 11-10

At Rehbach and Schladebach, the very basal part of the exposed fluvial units contains gravel and sand partly including reworked clay-rich and seldom organic deposits which are indicative of the onset of increased fluvial activity, including the reworking of warm-stage (Holsteinian) deposits during the MIS 11–MIS 10 transition. The dating-precision does not allow for the separation between MIS 11 or early MIS 10. However, an increased mobilization of gravel and corresponding aggradation is more likely to correspond to the climatic shift towards colder climatic periods^65^ than during an interglacial with presumably stabilized landscape surfaces.

The pIRIR_290_ luminescence age estimates obtained from the upper- and middle part of the section at Schladebach overlap within the error and are indicative of the aggradation of several meters of sand and gravel during MIS 10. Ice wedges inside the sedimentary sequence support the idea of fluvial activity at Schladebach under periglacial, cold climate conditions. The luminescence ages do not allow us to distinguish between various aggradation periods during MIS 10, but the ice wedges at different elevation levels suggest a periodically stable terrace surface. Generally, several fluvial aggradation events during MIS 10 can be assumed.

### MIS 9-6

MIS 9 sediments are not preserved at the investigated sites. The luminescence ages obtained from Rehbach and Zwenkau clearly show significant chronological gaps between the fluvial units. The most substantial hiatus was documented at the basal part of the Rehbach-section, where the approximately 400 ka old basal unit is capped by around 240 ka old deposits. This hiatus indicates significant erosion, most likely during MIS 8.

At Rehbach, Zwenkau and Markkleeberg the main periods of fluvial aggradation occurred between MIS 8 or the MIS 8 - MIS 7 transition to late MIS 6.

MIS 6 aggradation is shown at Rehbach, Zwenkau and Markkeeberg. The fluvial sequence at Rehbach represents the climatic shift from early- to late MIS 6 in the middle- to upper part of the sedimentary sequence. The deposits preserved at the middle part of the section, dated to 173 ± 16 ka show no permafrost features such as ice wedges whereas the upper part, dated to 144 ± 13 and 160 ± 13 ka, represents permafrost conditions towards the glacial maximum of MIS 6.

Fluvial aggradation in the study area may have been driven mainly by climatic shifts towards colder climate conditions during the Middle Pleistocene. The most rapid fluvial aggradation documented in this study occurred during MIS 10 and MIS 6.

### Interpretation of the ages for the Middle- and Lower Paleolithic stone artefact assemblages, and the implications for human dispersal into central Germany

The first hominin occupation of Europe and the human dispersal is discussed in several studies (^27,28,66,67^ among others), however evidence for the presence of sites older than 250 ka in central Europe is relatively sparse^27,28^. Therefore, the Schladebach/Wallendorf site, directly dated in the present study to MIS 11 or early MIS 10 (Figs 2; 4), adds an important data point to the ongoing debate about the human occupation and dispersal into central Europe. Another important site potentially attributed to the MIS 11 interglacial in central Germany^36^ is the Thuringian travertine site of Bilzingsleben^68–70^ (for a summary of the geology, the age estimations and the research history see the article by C. Pasda^35^). Unfortunately, ^230^Th/U dating on micro samples has thus far been unsuccessful, largely due to strong weathering of the travertine, resulting in open system behavior for U^71^. Nevertheless, because U series activity ratios yielded values close to the radioactivity equilibrium, it was suggested that the age of the site is likely ≥300 ka^71,72^. Although the anthropogenic origin of the majority of the artefacts has recently been questioned^35^, human cranial remains prove the presence of humans in Bilzingsleben^73^. A recent study of the megafauna from the site^74^ confirms the natural accumulation of many of the bones and also demonstrates ephemeral evidence for a human accumulation agent. We provide evidence here that Wallendorf/Schladebach, with more than 6700^32,33^ stone artefacts, confirms the presence of humans in central Germany around MIS 11 and/or early MIS 10. These humans had a Lower Paleolithic stone tool industry, with few MP features (e.g. prepared cores^32,33^) at that time.

Schöningen in Lower Saxony^75–77^ is the next younger Lower Paleolithic site confirming human presence in central Germany during the MIS 9 interglacial^34,36,78^ (Fig. 4). Although the MIS 9 ages based on ^230^Th/U dating on peat^78^ were rejected recently due to U-series open system behavior^19^, we favor the MIS 9 interpretation for the find layer of Schöningen based on: (1) the interpretation of the geological succession following the Elsterian sediments^36^, (2) a palynological record which is distinct from the Holsteinian^79^, and (3) the recently published MIS 9 ages based on luminescence dating of heated flint^34^. Besides exceptional faunal remains, like teeth and a humerus fragment of a saber-toothed cat, the site is famous for the preservation of wooden spears^75,80^. These thrusting spears demonstrate the manufacture and use of wooden hunting weapons in the Lower Paleolithic. Like in Schladebach/Wallendorf, the stone artefacts from Schöningen are attributed to the (late) Lower Paleolithic: prepared cores are not present, the tools and the blank production is based on rather thick and broad flakes and, although evidence for bifacial shaping is present, handaxes themselves are missing^81^.

During the following MIS 8, we have archaeological evidence in central Germany (Fig. 4) from Rehbach and Zwenkau. Previously, the lower find horizon of Markkleeberg was suggested to have an early MIS 8 age^49^ as well, but the IR-RF ages of M. Krbetschek (Fig. 2) indicate an MIS 7 age for the lower find horizon. The age we present for the base of the SMT in Zwenkau is suggestive of an onset of the MP in the region in early MIS 8, however, the relatively skewed De-distribution of sample L-Eva 1614 (overdispersion = 31%) may indicate an age-overestimation. Recent evidence from the site Kesselt-Op de Schans/Belgium also suggests an age for the Lower to Middle Paleolithic transition in Northwestern Europe during the end of MIS 9 and the onset of MIS 8, about 280 ka^31^. This matches the range of our age from Zwenkau. However, given the results of a recent comparative study about the early MP record in western and northern Europe^28^, there are only a few sites that have layers correlated with MIS 8, and there is little evidence for human presence during the MIS 8 Pleniglacial. The majority of the early MP sites in Europe are in fact associated with MIS 7^28,30^.

Considering this European evidence, an MIS 7 age for the occurrence of the fully developed MP in central Germany is more likely. This is underlined by the fact that the date for the basal SMT of Zwenkau overlaps in its lower error range with the MIS 7 age of Rehbach (which points to the MIS 8/ MIS 7 boundary), as well as the MIS 7 IR-RF ages for the basal find horizon of Markkleeberg. The hypothesis is reinforced by the Thuringian travertine site Weimar-Ehringsdorf. Several attempts^71,72,82^ have dated the formation of the travertine in Weimar-Ehringsdorf to MIS 7. The artefact assemblage found in several layers within the travertine of Weimar-Ehringsdorf is attributed to the Middle Paleolithic^83–85^. Importantly, several of the human skeletal remains have Neanderthal features^86^. Among them are the cranial remains of a female, and partial skeleton remains of a child^86^. The clearly identified Neanderthal features^87^ of the human remains indicate that with the emergence of the fully developed MP during MIS 8 (?) - MIS 7 in central Germany, the Neanderthal lineage was linked to this stone tool industry.

During the MIS 6 cold stage (Fig. 4), we have evidence from the upper find layer of Markkleeberg that humans adapted to the cold stage environment and were present in central Germany shortly before the major Saalian ice advance around 150 ka. The finds were described as sharp edged *in situ* artefacts, preserved in gravel lenses within small fluvial channels^49^. Therefore, we can infer that the finds were not reworked and/or transported from an older find horizon.

From the archaeological evidence in central Germany and the dates presented in this study, we can infer that:

1. The Lower Paleolithic human presence started in MIS 11 and lasted until about MIS 9.
2. Due to a significant chronological gap within the sedimentary units of the SMT, spanning from MIS 9-8, a reconstruction of human occupation during this time period is difficult. The basal age of Zwenkau (280 ± 45 ka) and the lower error range of the Rehbach basal date (239 ± 31 ka) point to human presence in the region of potentially early MIS 8. Furthermore, if we accept that the region was not inhabited during the MIS 8 glacial maximum, humans may have been present at the boundary of MIS 8 and MIS 7. With regard to the dates from sites like Markkleeberg and Ehringsdorf, we can infer that the bulk of MP sites dates to the beginning of MIS 7 in central Germany, at the latest.
3. The age of the upper find horizon in Markkleeberg is suggestive of human presence during the cold phases of MIS 6.

## Materials and Methods

### Materials

#### Schladebach/Wallendorf

The gravel pit of Schladebach, Saxony-Anhalt (51°18′22.66′′N, 12° 6′17.28′′E), is situated about 20 km west of Leipzig. Here, the SMT gravels of the Saale-Unstrut-river system are exploited by mining. About 3 km to the north are the former gravel pits of Wallendorf^32,33^. Here, more than 6700 artefacts were recovered from the base of the same SMT sequence and from the SMT coarse gravel dump of the pits. The artefacts were described as having a Lower Paleolithic character^32,33^. The flakes were detached from simple flake cores, with changing striking platforms and directions. Among the tools, notched pieces are the most numerous, followed by simple scrapers. Nevertheless, some MP traits were reported^32,33^: A small number of cores can be classified as prepared or Levallois cores (unfortunately no numbers are given in the cited publications), and some flakes show facetted platforms. Additionally, some bifacially worked tools, like simple handaxe-like forms, occur in the assemblage. During a survey at the exposed SMT base at the Schladebach pit, which was conducted by the authors in addition to the luminescence sampling, 10 cores (Supplementary Figures S23–S25), 16 flakes, 2 scrapers, and a potentially notched tool (Fig. 3A) were recovered (see also Supplementary Tables S3 and S4.

The luminescence samples were taken from the exposed SMT sequence of the ongoing gravel pit.

#### Markkleeberg

Markkleeberg (51°16′15.29′′N, 12°24′5.35′′E), Saxony, is situated about 8 km south of the center of Leipzig, and is one of the most important sites for the early MP in Germany. The first artefacts were found at the end of the 19^th^ and the beginning of the 20^th^ century^88^ in several gravel pits within the SMT sequence (Pleiße/Gösel-river system). These finds were comprehensively published by Grahmann in 1955^48^. Later, at the end of the 1970s until the beginning of the 1980s, about 4500 artefacts were recovered and partly excavated from the base of the fluvial sequence^47^. Archaeological surveys were carried out as accompanying measures to the open-cast brown coal mining, which took place east of the town of Markkleeberg and affected the formerly described find locations of the artefacts. The last excavations in Markkleeberg took place between 1999 and 2001^49^, when the mine was continuously flooded to create a lake. From this recent excavation, three find horizons were reported:^49^ (1) the main find horizon at the base of the terrace sequence with partly *in situ* find concentrations, where also the artefacts found between 1977–1980^47^ were recovered, (2) the middle find horizon, with rolled, re-deposited artefacts, and (3) an upper find horizon at the top of the SMT sequence with sharp-edged, *in situ* artefacts, corresponding to the layers were the artefacts were recovered at the beginning of the 20^th^ century^49^ (pit “Markleeberg d”). The artefact assemblages from Markkleeberg can be characterized as typical MP, with prepared or Levallois cores, scrapers and bifacial tools such as handaxes^47–49^.

The site of Markkleeberg is no longer accessible. The luminescence samples were taken by M. Krbetschek during fieldwork campaigns between 1999 and 2001, and were made accessible to us by the Freiberg (Saxony) dating laboratory.

#### Zwenkau

The MP artefacts were recovered in a former brown coal open-cast mine named Zwenkau, Saxony (51°14′11.68′′N, 12°16′11.88′′E), which is approximately 14 km south-east of the center of Leipzig. Within the open-cast mine, the SMT sequence of the Elster-river system was accessible. Because of villages situated formerly in the area of the mine, the artefacts are published under the site names “Eythra” and “Bösdorf”^50^ (Fig. 3C). 1850 artefacts^50^ were recovered during surveys of the base of the SMT. The high proportion of bifacial tools is a particularly significant feature of this collection. About 50% of all tools are bifaces such as handaxes, knives and bifacial scrapers. Levallois cores are present, but are not very common. The majority of the cores are opportunistic flake cores, with several exploition surfaces (pers. comm. W. Bernhardt, Schkeuditz, who recovered and documented the artefacts).

The luminescence samples derive from a part of the SMT sequence which is preserved in the western slope of the former open-cast brown coal mine. In this area, a bifacial scraper was found in the profile of the basal part of the SMT (Fig. 3C, pers. comm. W. Bernhardt, November 20^th^ 2017, who recovered and documented the artefact).

#### Rehbach

The gravel pit of Rehbach, Saxony (51°16′11.43′′N, 12°16′59.46′′E), is situated approximately 4 km north-west of the former coal mine Zwenkau, and is situated within the gravels of the same SMT (Elster-river system) sequence. During sampling for luminescence dating, we recovered a typical MP Levallois core and a flake (Fig. 3B) from dislocated SMT basal gravels. Another flint flake was found in stratigraphic position within the SMT sequence (Supplementary Figure S27).

The luminescence samples were taken from the exposed SMT sequence of the ongoing gravel pit.

## Methods

### Luminescence sample preparation and instrumental details

Luminescence-samples and material for gamma spectrometry were taken at equal positions. Sample preparation followed the common steps, including the removal of carbonates and organic matter in HCl and H_2_O_2_. To separate feldspar from heavy minerals and quartz, either density separation using lithium heterotungstate, or, for samples from Markkleeberg and Zwenkau, the flotation technique^89^ was used. All samples from the section Markkleeberg were etched for 40 min using 10% HF to remove the alpha-ray affected outer rim of the coarse feldspar grains.

Finally, the sample material was mounted on steel discs (aliquots) using silicon spray.

Equivalent dose (D_e_) measurements were undertaken using automated Risø TL-DA-20 reader. The feldspar signal was stimulated using IR light-emitting diodes transmitting at 870 nm (145 mW/cm^2^) and the feldspar signal was detected in the blue-violet wavelength region. Irradiation was provided by a calibrated ^90^Sr/^90^Y beta source with a dose-rate of ~0.24 Gy/s.

### Dose rate determination

Dose rates were determined based on high resolution germanium gamma spectrometric analysis of the activities of uranium, thorium, potassium, and their daughter isotopes. All samples were measured on the bulk material at the “Felsenkeller” laboratory (VKTA) in Dresden.

Dose rate attenuation by moisture was accounted for using water content values of 15 ± 10%. The cosmic-dose rate contribution to the ionization of minerals was based on Presscot and Hutton^90^.

The internal potassium content was assumed to be at 12.5 ± 0.5%^91^.

To account for alpha efficiency an a-value of 0.11 ± 0.02 ^92^was used for non-etched samples and the dose rate conversion factors were taken from Guerin *et al*.^93^.

### Equivalent dose measurements

Luminescence dating was applied to coarse-grained K-feldspar using the pIRIR_290_ approach similar to Thiel *et al*.^45^. Prior to the detection of the elevated temperature feldspar signal at 290 °C for 200 seconds, the IR_50_ signal, mostly affected by higher rates of anomalous fading, was depleted by stimulating with infrared-diodes for 100 seconds. The measurement protocol is shown in Supplementary Table S1. The pIRIR_290_ approach was chosen due to its high signal stability (negligible fading) and as its suitability to date Middle Pleistocene sediments^46^.

For equivalent dose measurements, grain size fractions of 180–250 microns were used for material from the sites Rehbach, Zwenkau and Schladebach/Wallendorf.

For the samples from Markkleeberg, the 90–160 microns fraction was used.

All equivalent dose measurements were conducted using very small aliquots with a sample-diameter between 0.5 mm–1 mm. Hence, only a very few grains were put on one aliquot allowing to point out if there was any skewness in equivalent dose distribution due to insufficient bleaching.

For each sample, 3–5 artificial doses were inserted to create the dose response curve.

At the end of the measurement cycle of the pIRIR_290_ SAR approach, the first artificial dose was inserted again to measure the recycling ratio as quality criteria. For final equivalent dose estimation, only aliquots yielding recycling ratios deviating within 10% from unity were accepted.

Equivalent dose overdispersions are mostly >25% (see Supplementary Table S2) and equivalent dose distributions indicate mostly sufficient bleaching (see Supplementary Figures S1–S18). Therefore the Central Age Model^94^ was used for age calculations.

Dose recovery tests were conducted on samples L-Eva 1594, 1599, 1600 and 1612. Therefore, the sample material was bleached for 24 hrs under a solar simulator (UVA cube 400). Remaining dose residuals were then measured for respectively 3 aliquots from each sample. A further 3 aliquots were irradiated with a known dose close to the assumed natural one, and afterwards the precision of the recovered dose was tested. All residual subtracted measured to given dose ratios are within ±10% deviation from unity and are at 1.08 ± 0.04 (L-Eva 1594), 1.09 ± 0.07 (L-Eva 1599), 0.93 ± 0.22 (L-Eva 1600) and 1.09 ± 0.02 (L-Eva 1612).

Fading measurements following Huntley and Lamothe^95^ were conducted on 6 aliquots from samples L-Eva 1594, L-Eva 1612 and L-Eva 1638. The pIRIR_290_ g-values are at 2.4 ± 0.37, 1.53 ± 0.59 and 1.37 ± 0.20 respectively.

The presented ages are non-fading corrected as obtained pIRIR_290_ fading rates are low and fading is interpreted to have only negligible effect on the equivalent doses.

Additionally, pIRIR_225_ measurements^43,44^ were conducted on samples L-Eva 1594 and L-Eva 1597 (Rehbach gravel pit). The pIRIR_225_ ages are presented and discussed in the supplementary part of the paper.

### Data Availability

The datasets generated during the current study are available from the corresponding author on reasonable request.

## Electronic supplementary material

Supplementary Information
