# Supplementary material for: Timing of the Saalian- and Elsterian glacial cycles and the implications for Middle – Pleistocene hominin presence in central Europe
Source: Sci Rep. 2018 Mar 23;8:5111. doi: 10.1038/s41598-018-23541-w (PMC5865135; doi:10.1038/s41598-018-23541-w)
Supplement: Supplementary file 1 — Supplementary Information [file 41598_2018_23541_MOESM1_ESM.pdf]

Supplementary Information

Timing of the Saalian- and Elsterian glacial cycles and the implications for Middle – Pleistocene hominin presence in central Europe

Tobias Lauer[1] *& Marcel Weiss*[1]

[1]*Max-Planck-Institute for Evolutionary Anthropology, Department of Human Evolution; Deutscher Platz 6, D-04103 Leipzig, Germany*

Chapter I – Luminescence Dating

Supplementary Table S1: Used measurement protocol.

Step	Treatment	Observed
1	Give dose, D_i → irradiation of the sample	
2	Preheat at 320 °C, 60 s	
3	IR stimulation at 50°C, 100 s	
4	IR stimulation at 290 °C, 200 s	Lx
5	Give test dose, D_t (irradiation of the sample)	
6	Preheat at 320 °C, 60 s	
7	IR stimulation at 50°C, 100 s	
8	IR stimulation at 290°C, 200 s	Tx
9	Return to step 1	

Supplementary Table S2: Table including dose rate data and obtained equivalent doses.

Abbreviations: DR = dose rate; De = equivalent dose (with standard-error); CAM = Central age model; Nr. al. = number of accepted aliquots.

Site	Sample ID	U (ppm)	Th (ppm)	K (%)	DR cosmic (mGy/ a)	DR total (mGy/a)	De (Gy) CAM $pIRIR_{290}$	$pIRIR_{290}$ age (ka)	Nr. al.
Schladebach	L-Eva 1622	0.66 ± 0.14	2.40 ± 0.16	0.98 ± 0.10	0.07 ± 0.01	1.72 ± 0.20	770 ± 21	447 ± 52	26
Schladebach	L-Eva 1725	0.80 ± 0.18	3.05 ± 0.20	1.24 ± 0.13	0.07 ± 0.01	1.88 ± 0.20	817 ± 32	406 ± 44	17
Schladebach	L-Eva 1724	0.61 ± 0.12	2.57 ± 0.19	1.28 ± 0.08	0.07 ± 0.01	1.98 ± 0.19	766 ± 39	387 ± 42	23
Schladebach	L-Eva 1599	0.71 ± 0.17	2.83 ± 0.19	1.39 ± 0.09	0.09 ± 0.01	2.13 ± 0.19	730 ± 58	343 ± 42	15
Schladebach	L-Eva 1600	0.82 ± 020	3.48 ± 0.24	1.40 ± 0.11	0.12 ± 0.01	2.23 ± 0.20	805 ± 27	360 ± 34	24
Schladebach	L-Eva 1601	0.94 ± 021	3.26 ± 0.23	1.88 ± 0.16	0.16 ± 0.02	2.68 ± 0.21	907 ± 39	338 ± 31	11
Rehbach	L-Eva 1621	0.52 ± 0.16	2.16 ± 0.17	1.21 ± 0.10	0.07 ± 0.01	1.88 ± 0.20	794 ± 34	423 ± 48	28
Rehbach	L-Eva 1626	0.64 ± 0.13	2.50 ± 0.18	1.23 ± 0.09	0.07 ± 0.01	1.94 ± 0.19	750 ± 54	387 ± 48	15
Rehbach	L-Eva 1627	0.81 ± 0.14	2.02 ± 0.14	0.88 ± 0.07	0.07 ± 0.01	1.64 ± 0.19	392 ± 23	239 ± 31	22
Rehbach	L-Eva 1722	0.78 ± 0.18	3.10 ± 0.2	1.71 ± 0.17	0.10 ± 0.01	2.44 ± 0.22	421 ± 08	173 ± 16	24
Rehbach	L-Eva 1597	1.42 ± 0.21	4.80 ± 0.30	2.09 ± 0.17	0.14 ± 0.01	3.03 ± 0.22	485 ± 18	160 ± 13	21
Rehbach	L-Eva 1594	1.16 ± 0.18	3.38 ± 0.20	1.87 ± 0.19	0.14 ± 0.01	2.70 ± 0.22	390 ± 15	144 ± 13	19
Zwenkau	L-Eva 1614	0.58 ± 0.18	1.43 ± 0.10	0.47 ± 0.04	0.10 ± 0.01	1.25 ± 0.19	349 ± 21	280 ± 45	26
Zwenkau	L-Eva 1613	1.03 ± 0.25	3.23 ± 0.20	0.81 ± 0.06	0.11 ± 0.01	1.75 ± 0.19	323 ± 15	185 ± 22	23
Zwenkau	L-Eva 1612	0.90 ± 0.30	2.70 ± 0.20	0.97 ± 0.08	0.14 ± 0.01	1.85 ± 0.19	338 ± 14	183 ± 21	24
Markkleeberg	L-Eva 1640	1.05 ± 0.02	3.74 ± 0.17	1.06 ± 0.01	0.12 ± 0.01	1.96 ± 0.18	426 ± 24	217 ± 24	22
Markkleeberg	L-Eva 1637	0.67 ± 0.02	2.51 ± 0.12	1.17 ± 0.01	0.14 ± 0.01	1.93 ± 0.18	308 ± 16	159 ± 17	17
Markkleeberg	L-Eva 1638	0.77 ± 0.02	2.92 ± 0.13	1.16 ± 0.01	0.15 ± 0.01	1.97 ± 0.18	324 ± 09	164 ± 16	21

Comment on conducted fading measurements:

As outlined in the methods chapter, g-values obtained from samples L-Eva 1594, L-Eva 1612 and L-Eva 1638 are at 2.4 ± 0.37, 1.53 ± 0.59 and 1.37 ± 0.20 respectively. The corresponding IR_{50} g-values are at 5.51 ± 1.77, 3.56 ± 1.36 and 2.69 ± 0.52.

Recuperation:

All accepted aliquots yielded a recuperation < 5 %:

De-scatter plots including CAM (Central Age Model) based De-values (red bars).

Schladebach/ Wallendorf

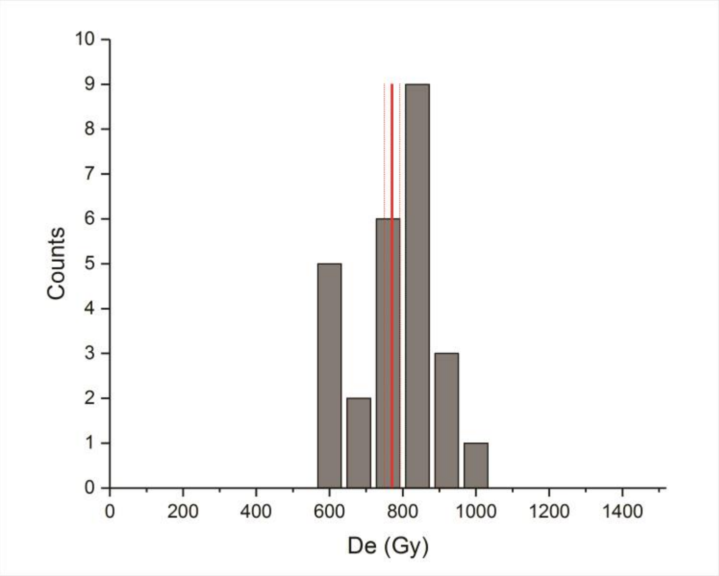

Supplementary Figure S1. Sample L-Eva 1622, Schladebach/ Wallendorf; OD (Overdispersion) = 13 %.

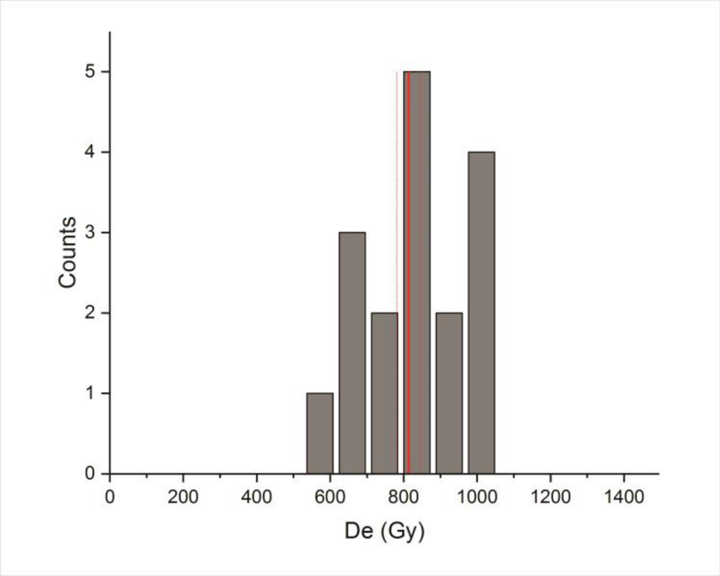

Supplementary Figure S2. Sample L-Eva 1725, Schladebach/ Wallendorf; OD = 15 %.

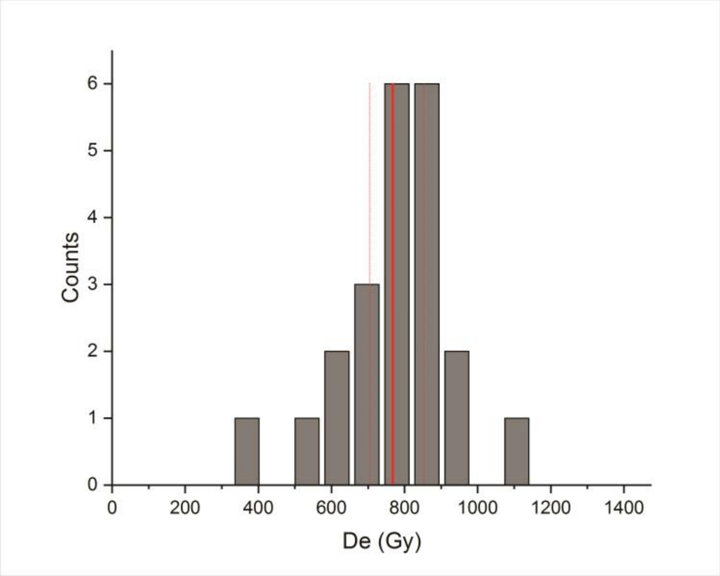

Supplementary Figure S3. Sample L-Eva 1724, Schladebach/ Wallendorf; OD = 24 %.

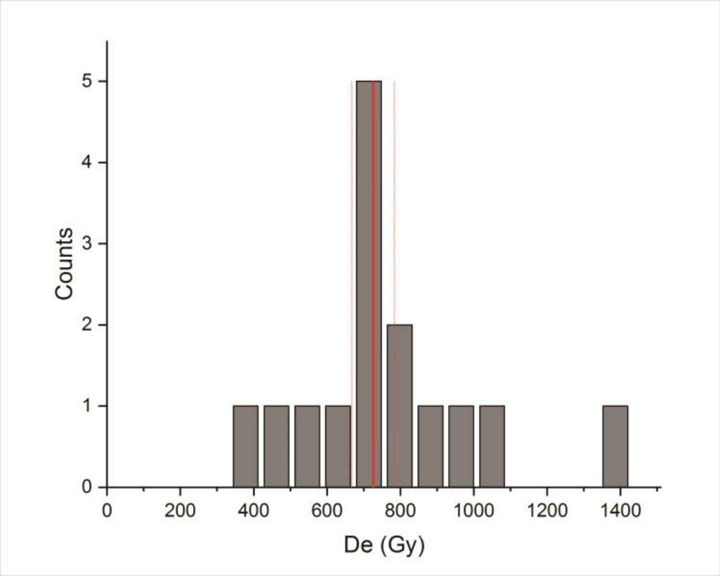

Supplementary Figure S4. Sample L-Eva 1599, Schladebach/ Wallendorf; OD = 30 %.

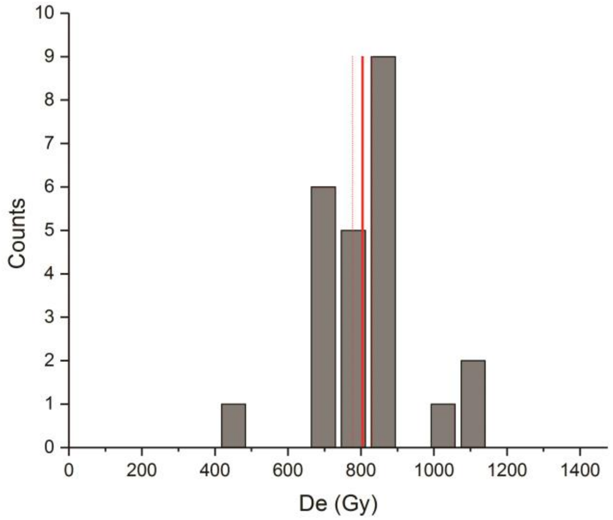

Supplementary Figure S5. Sample L-Eva 1600, Schladebach/ Wallendorf; OD = 16 %.

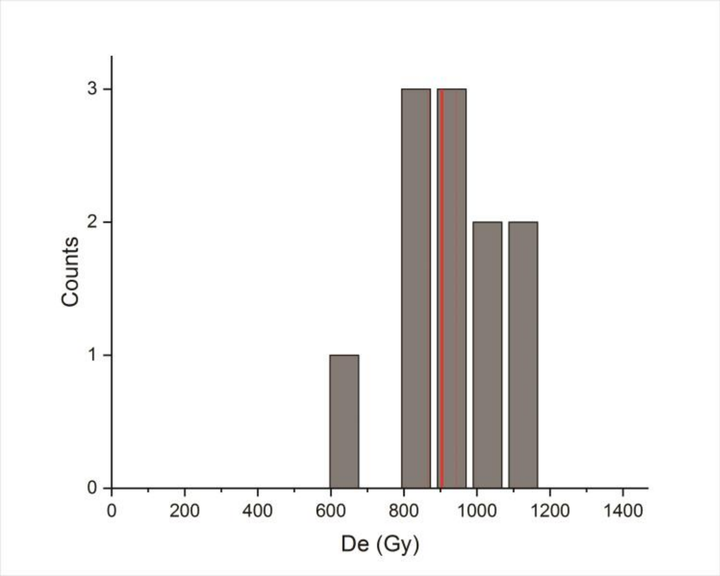

Supplementary Figure S6. Sample L-Eva 1601, Schladebach/ Wallendorf; OD = 13 %.

Rehbach

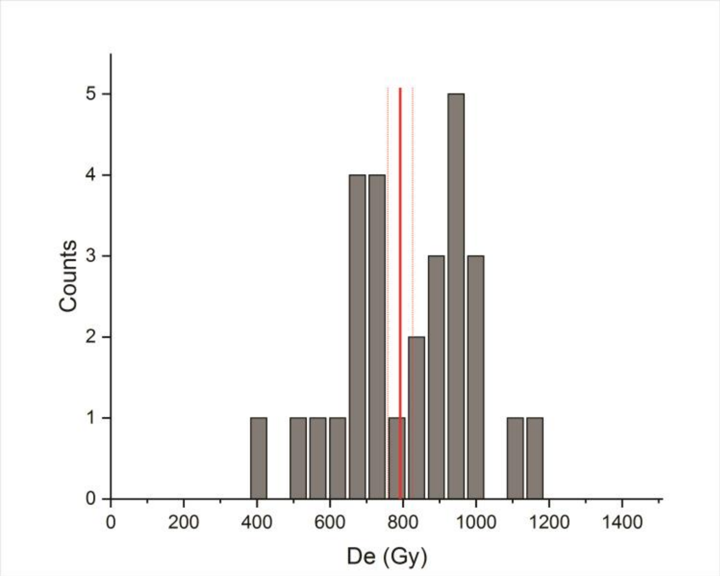

Supplementary Figure S7. Sample L-Eva 1621, Rehbach; OD = 22 %.

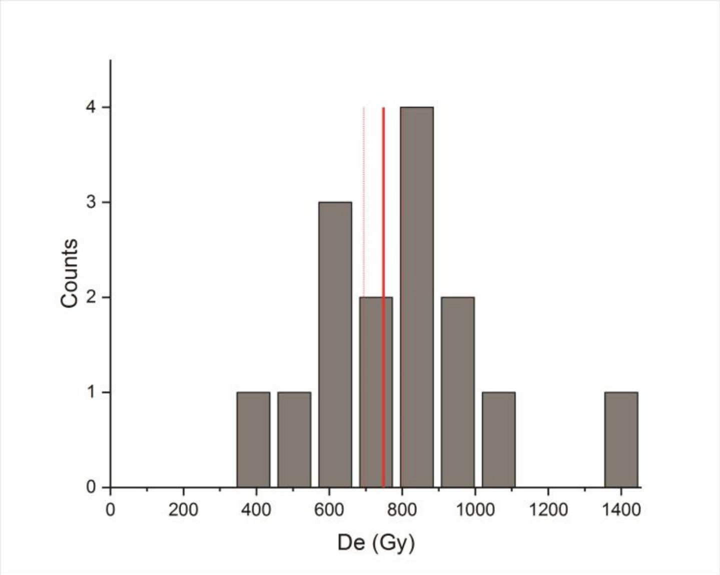

Supplementary Figure S8. Sample L-Eva 1626, Rehbach; OD = 27 %.

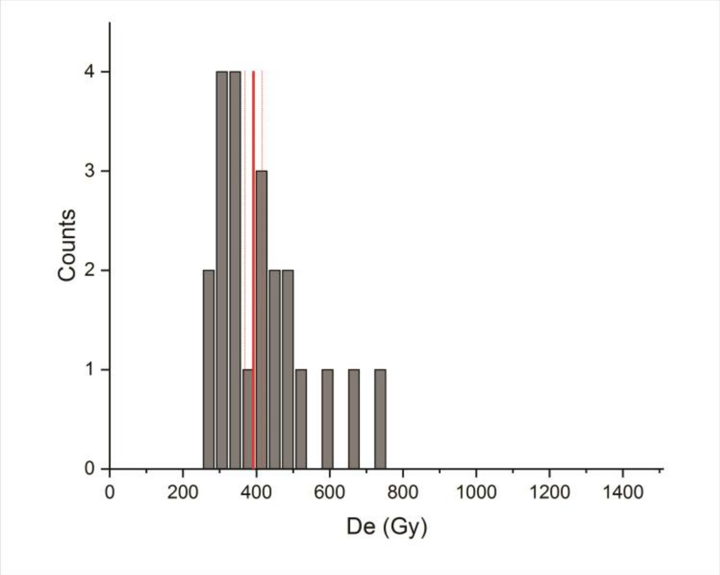

Supplementary Figure S9. Sample L-Eva 1627, Rehbach; OD = 28 %.

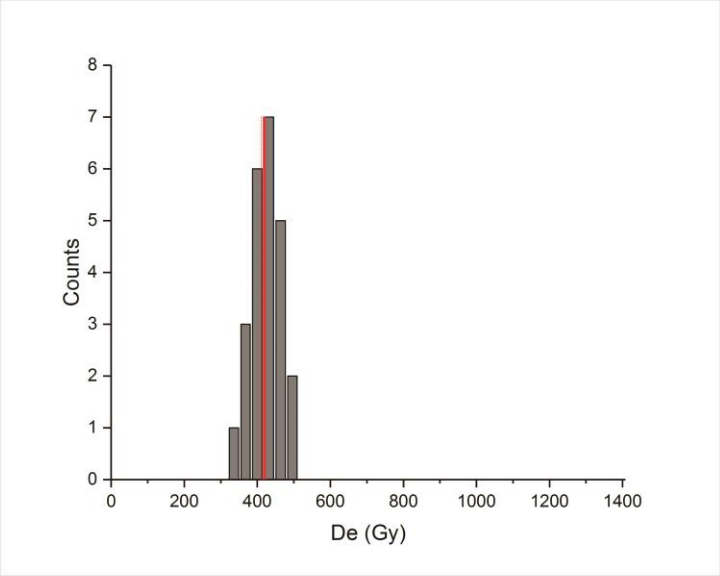

Supplementary Figure S10. Sample L-Eva 1722, Rehbach; OD = 24 %.

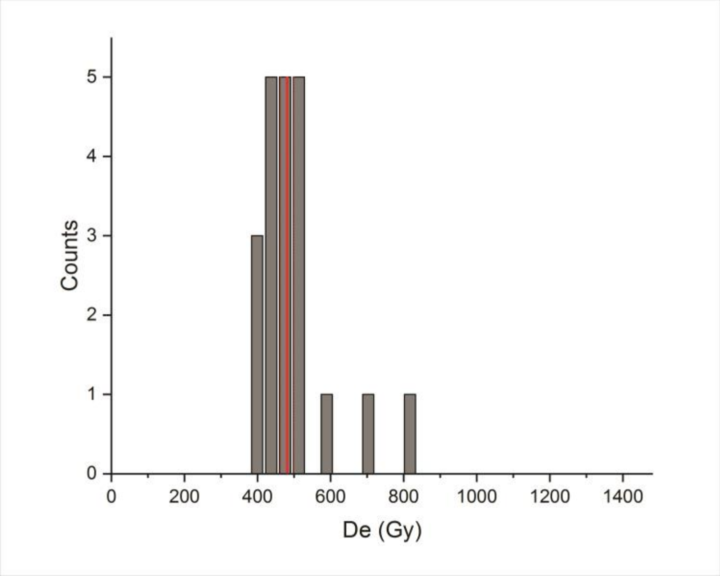

Supplementary Figure S11. Sample L-Eva 1597, Rehbach; OD = 16 %.

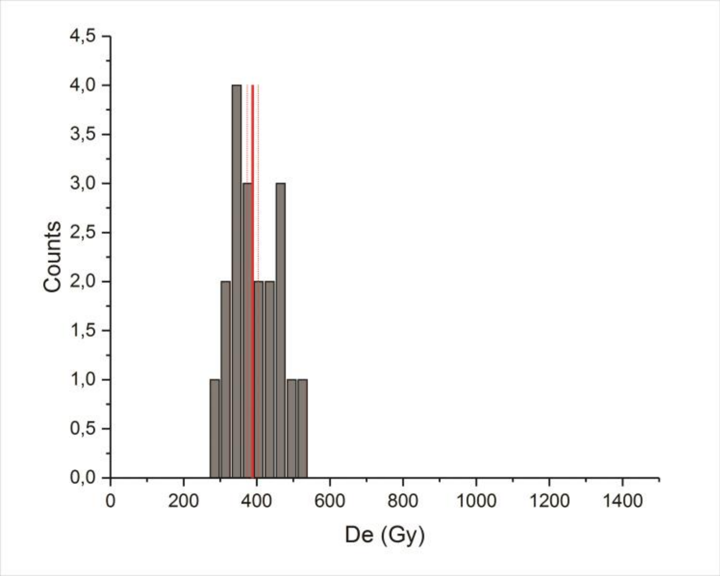

Supplementary Figure S12. Sample L-Eva 1594, Rehbach; OD = 16 %.

Zwenkau

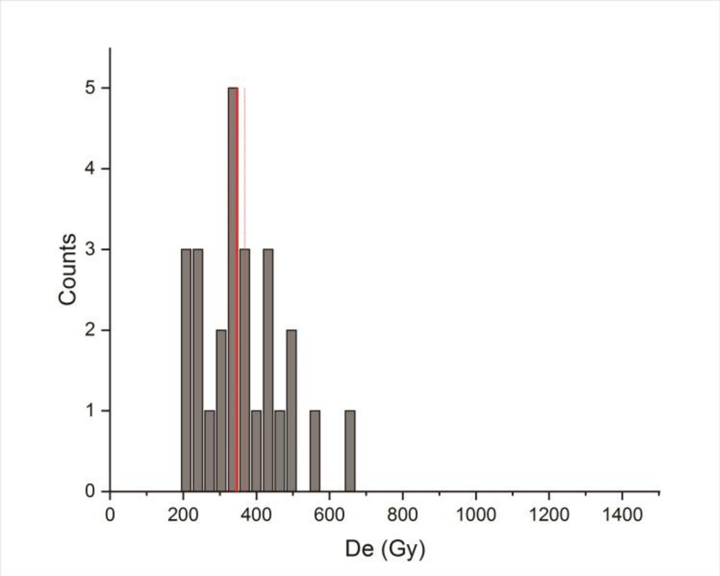

Supplementary Figure S13. Sample L-Eva 1614, Zwenkau; OD = 31 %.

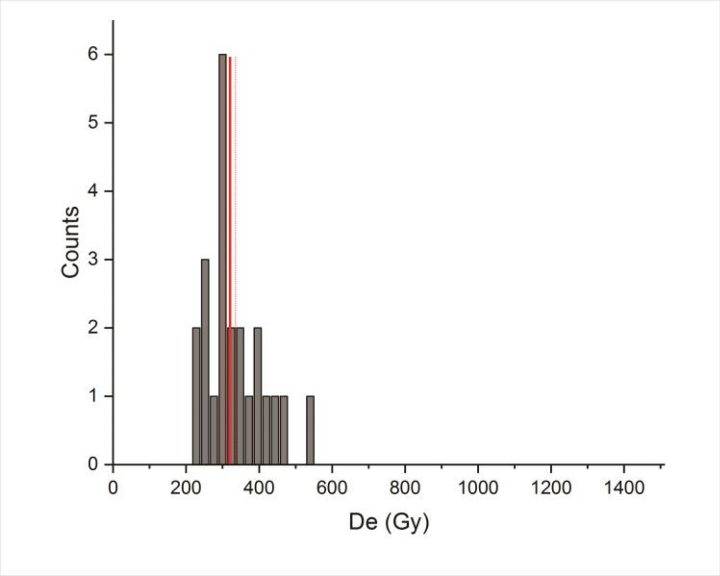

Supplementary Figure S14. Sample L-Eva 1613, Zwenkau; OD = 22 %.

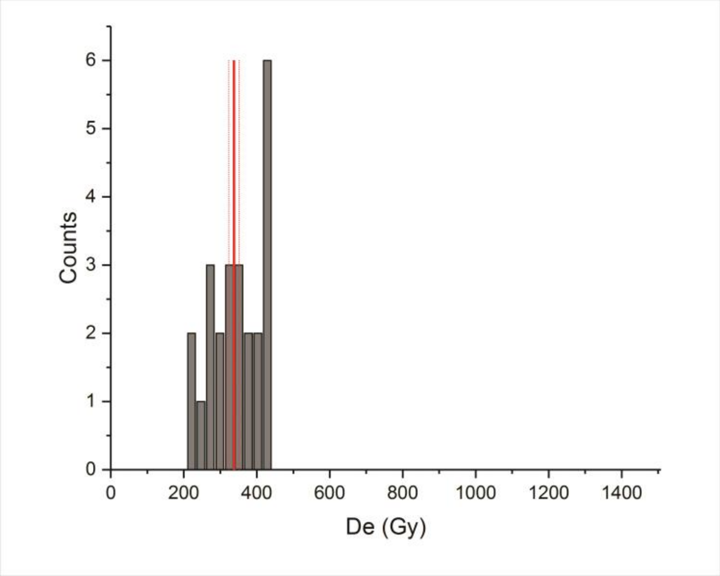

Supplementary Figure S15. Sample L-Eva 1612, Zwenkau; OD = 20 %.

Markkleeberg

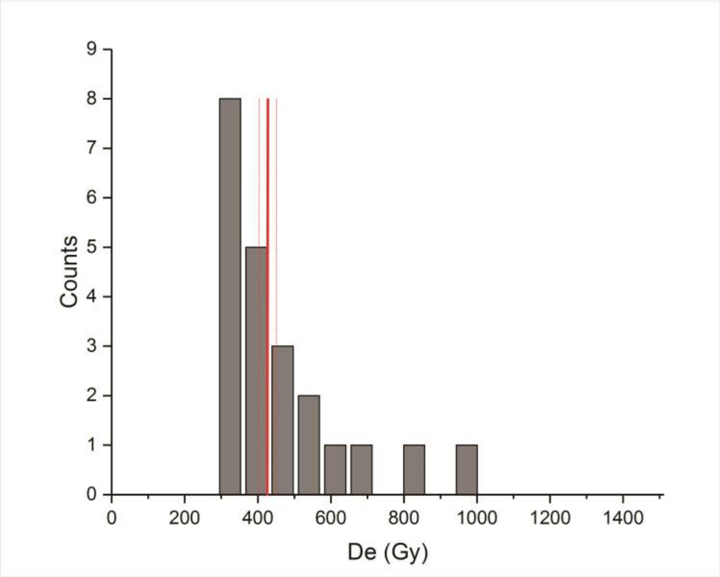

Supplementary Figure S16. Sample L-Eva 1640, Markkleeberg; OD = 25 %.

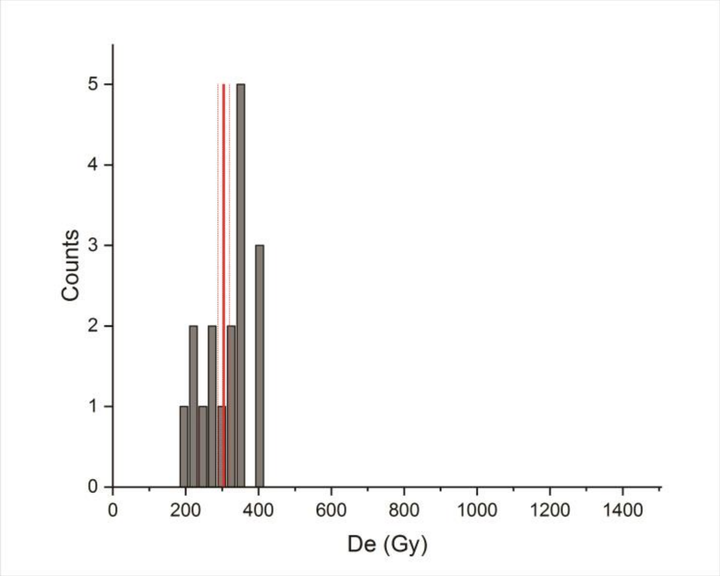

Supplementary Figure S17. Sample L-Eva 1637, Markkleeberg; OD = 20 %.

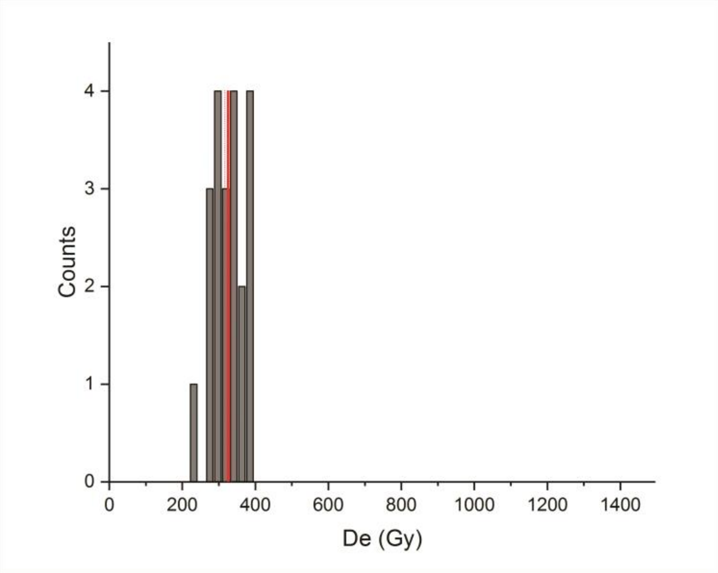

Supplementary Figure S18. Sample L-Eva 1638, Markkleeberg; OD = 13 %.

pIRIR$_{225}$-dating

Additionally to the used pIRIR$_{290}$-approach, the pIRIR$_{225}$ protocol was tested for its suitability on samples L-Eva 1594 and L-Eva 1597 (upper part of the SMT exposed at Rehbach). Here, the luminescence signal is stimulated at 225°C after depleting the IRSL signal at 50°C. Preheat- and cutheat temperatures were set to 250°C and hold for respectively 60 seconds. IR-stimulation at 50°C was conducted for 100 seconds and the pIRIR$_{225}$-signal was recorded for respectively 200 seconds. At the end of each measurement-cycle, an IR-cleanout for 40 seconds at 290°C was inserted.

For equivalent dose measurements, respectively 24 aliquots were used. The central age model based pIRIR$_{225}$ ages from samples L-Eva 1594 and L-Eva 1597, taken from the uppermost SMT unit (below the Saalian till) are at 102 ± 9 ka and106 ± 8 ka. The corresponding equivalent doses are 275 ± 5 Gy (L-Eva 1594) and 321 ± 11 Gy (L-Eva 1597). The stratigraphical position below the Saalian till (pre-Eemian and pre-Drenthe) shows that the non-fading corrected pIRIR$_{225}$ age estimates are underestimated. That highlights that using the pIRIR$_{225}$-approach requires fading corrections.

That supported, that the usage of the pIRIR$_{290}$-approach, for which only negligible fading can be assumed, seems most suitable for the investigated samples.

Chapter II – Artefact Surveys, additional Artefacts and Stratigraphy

1. Schladebach

In the following, we like to present some photographs of our main survey in the gravel pit Schladebach, Mai 17th, 2017. The survey took place at the exposed basal part of the Saalian Main terrace (SMT). Additionally, pictures of three cores will be presented here (Fig. S23-S25).

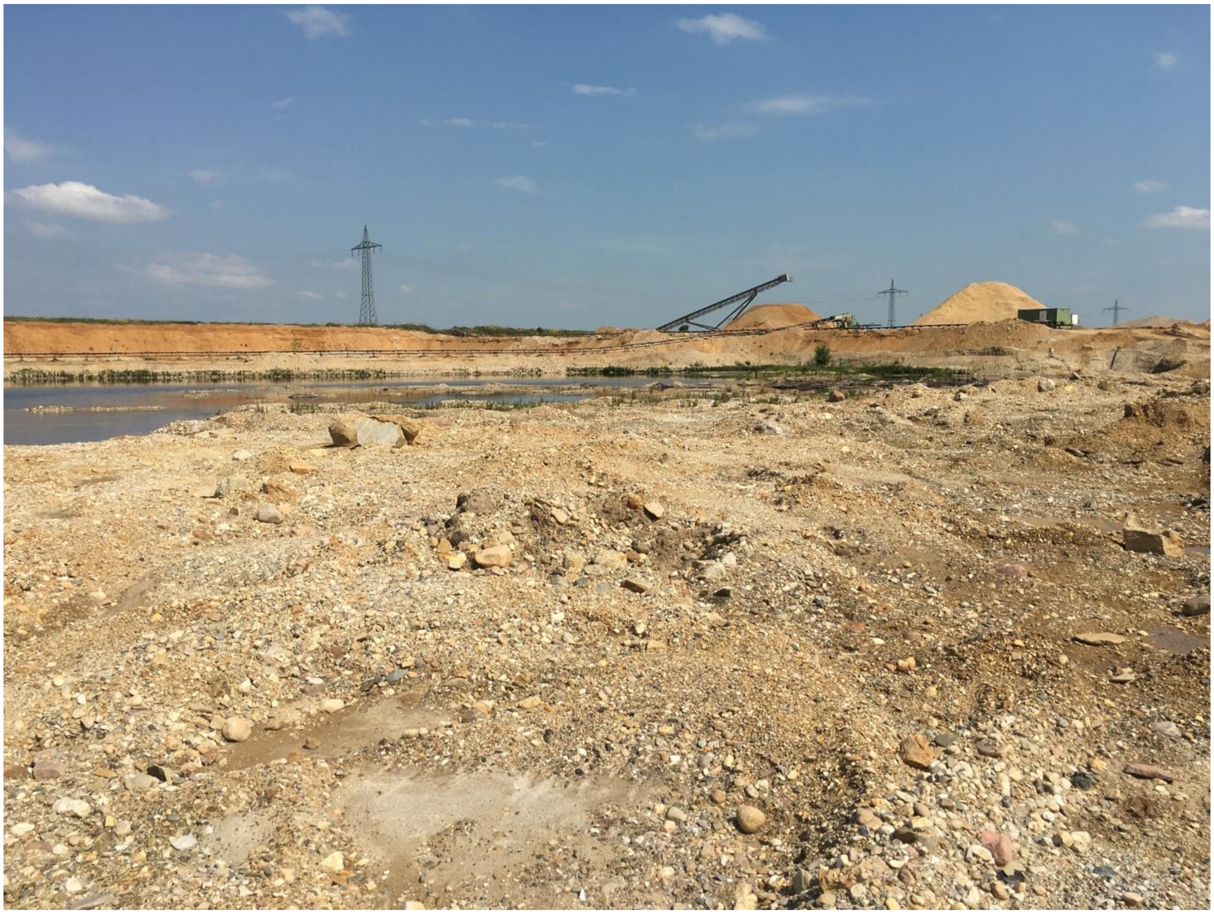

Supplementary Figure S19. The exposed basal part of the SMT in Schladebach. Note the large blocks, which were out washed by the river from the second Elsterian moraine. Photo: M. Weiss.

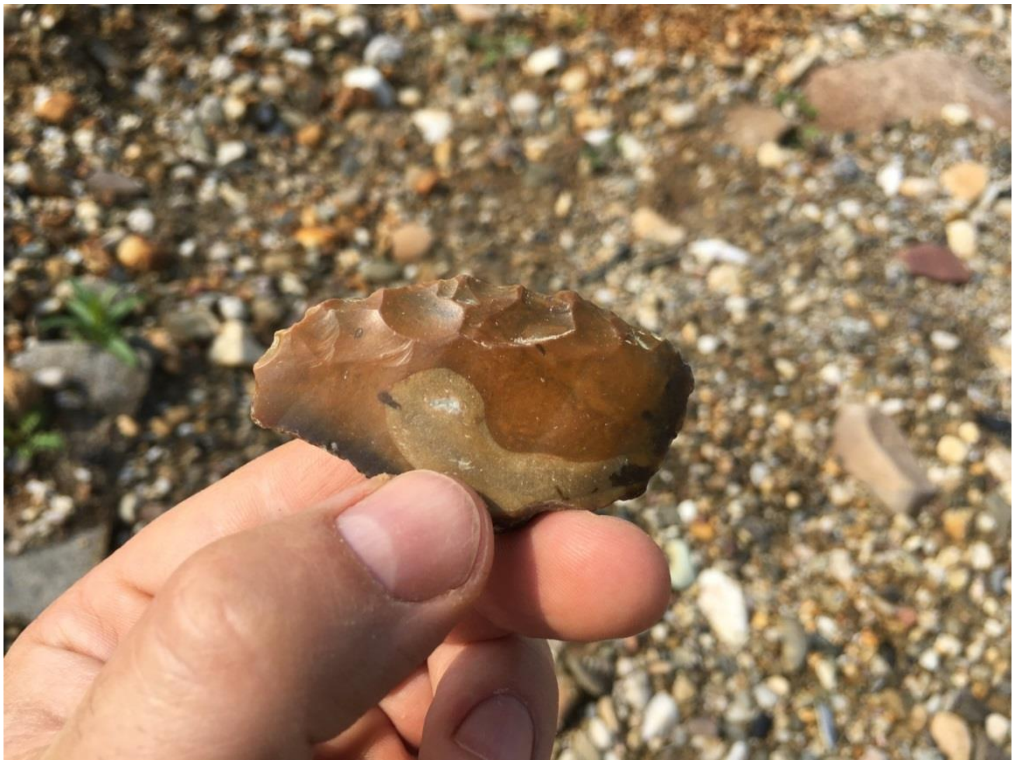

Supplementary Figure S20. Scraper (11263:1000:8) found within the basal gravels in Schladebach. Photo: M. Weiss.

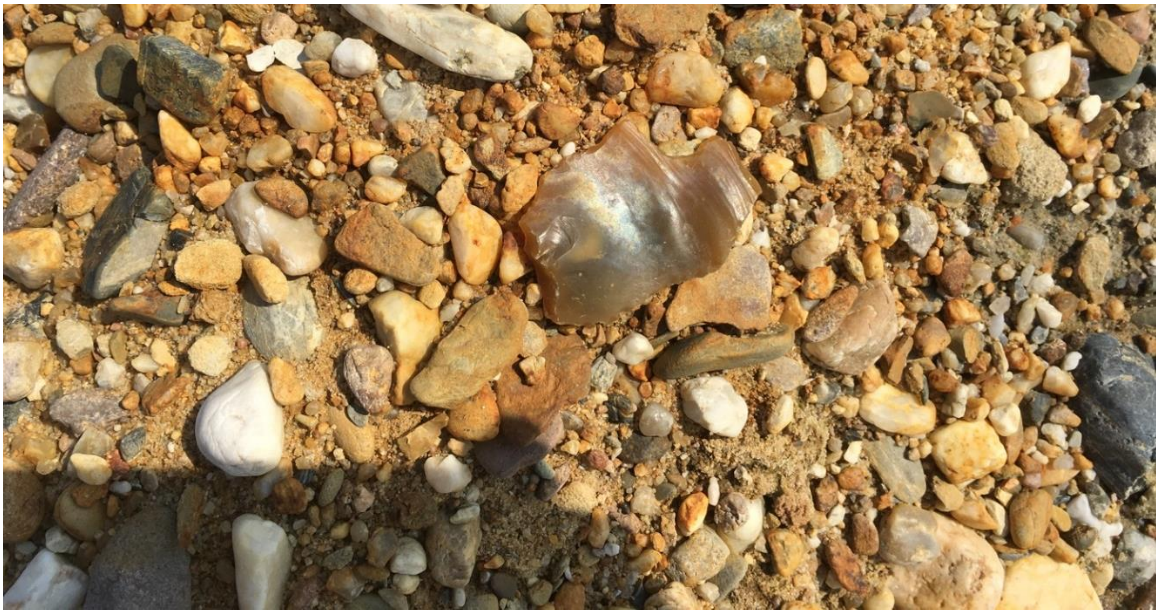

Supplementary Figure S21. Flake (ventral facing up, in the center of the photograph; 11263:1000:1) in in situ position within the basal gravels of the SMT in Schladebach. Photo: M. Weiss.

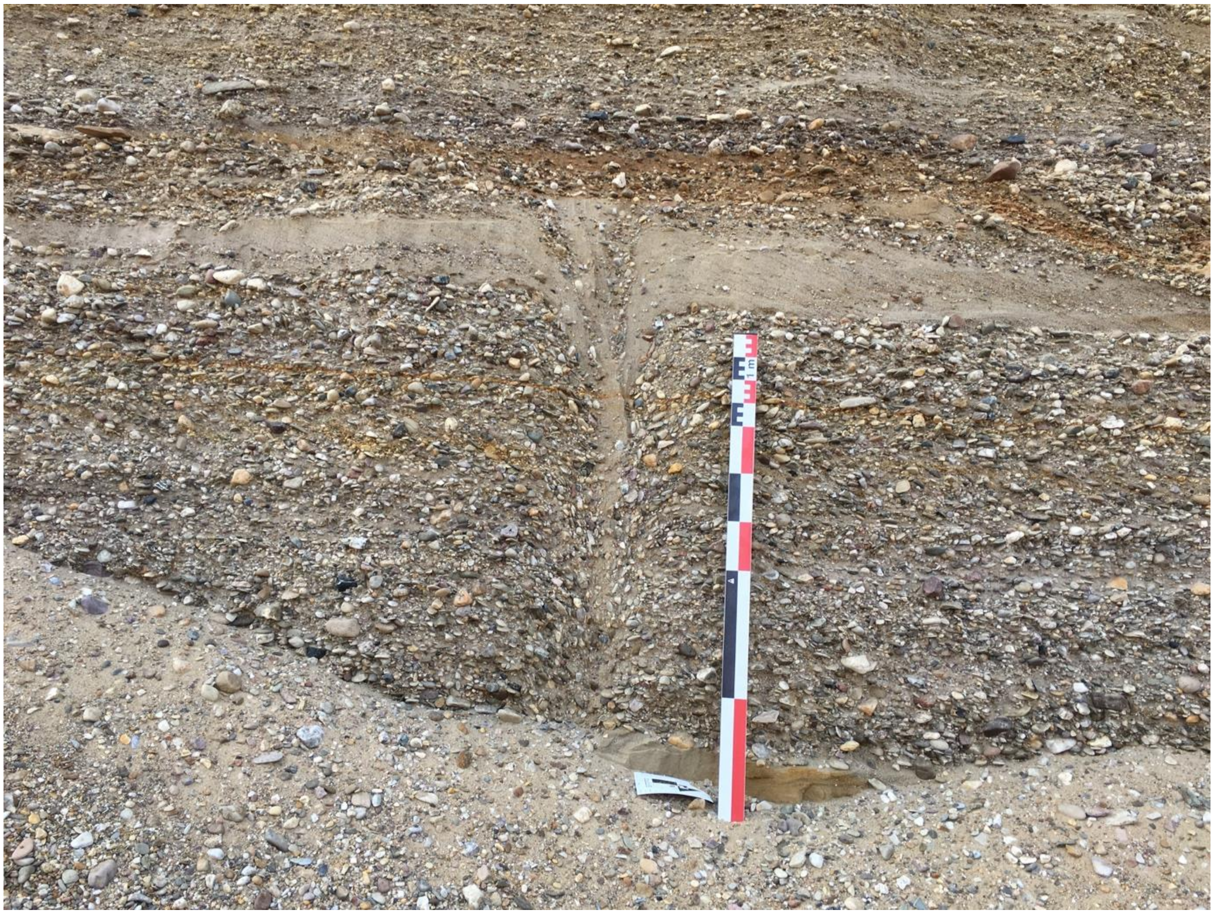

Supplementary Figure S22. Ice wedge pseudomorphism in the middle to upper part of the SMT in Schladebach, suggesting that the middle to upper part of the sequence was deposited during periglacial conditions (February 2nd, 2017). Photo: M. Weiss.

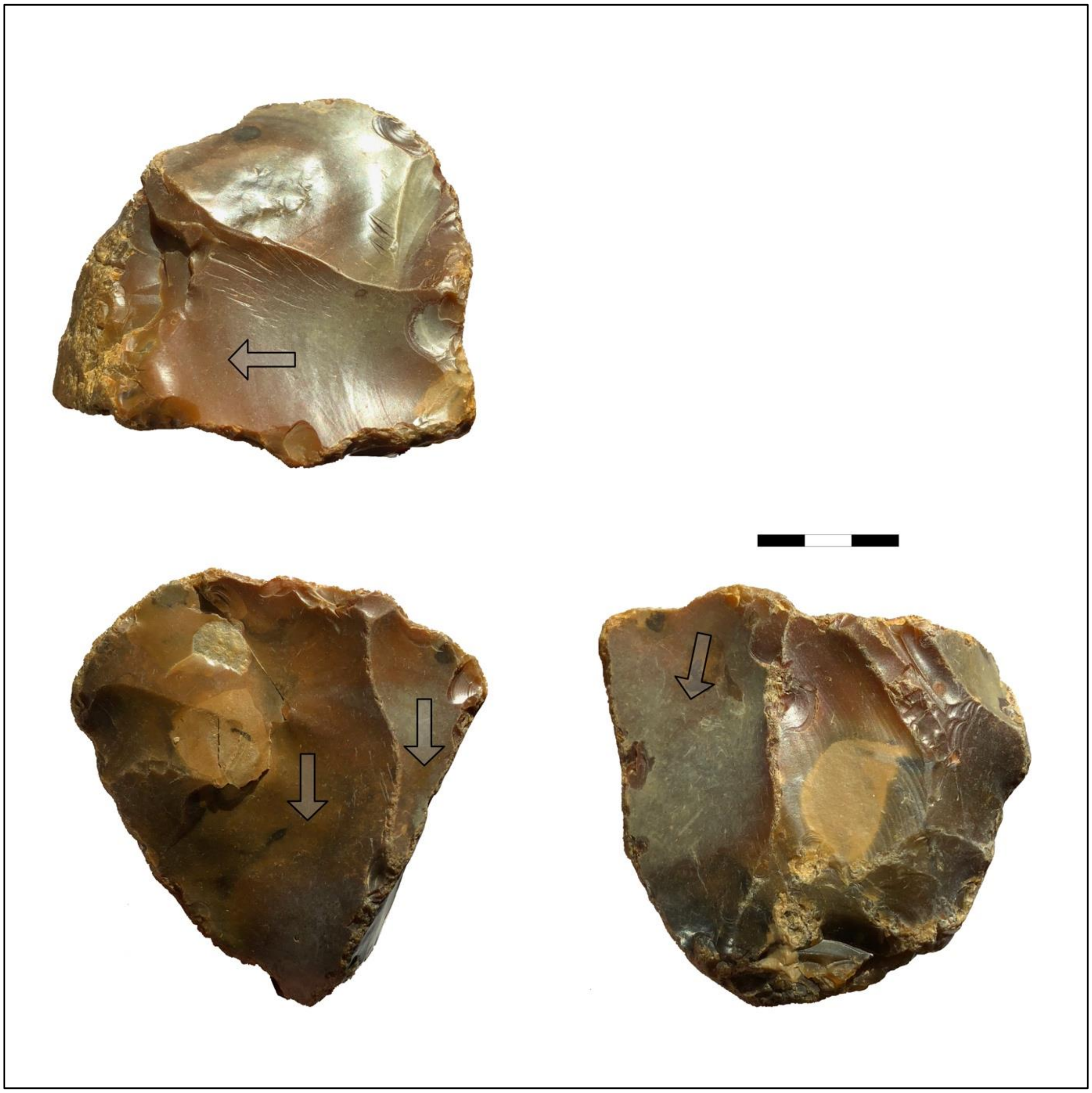

Supplementary Figure S23. Rolled, simple flake core (11263:1000:30) with three exploiting surfaces found in the Schladebach pit. Photo: M. Weiss.

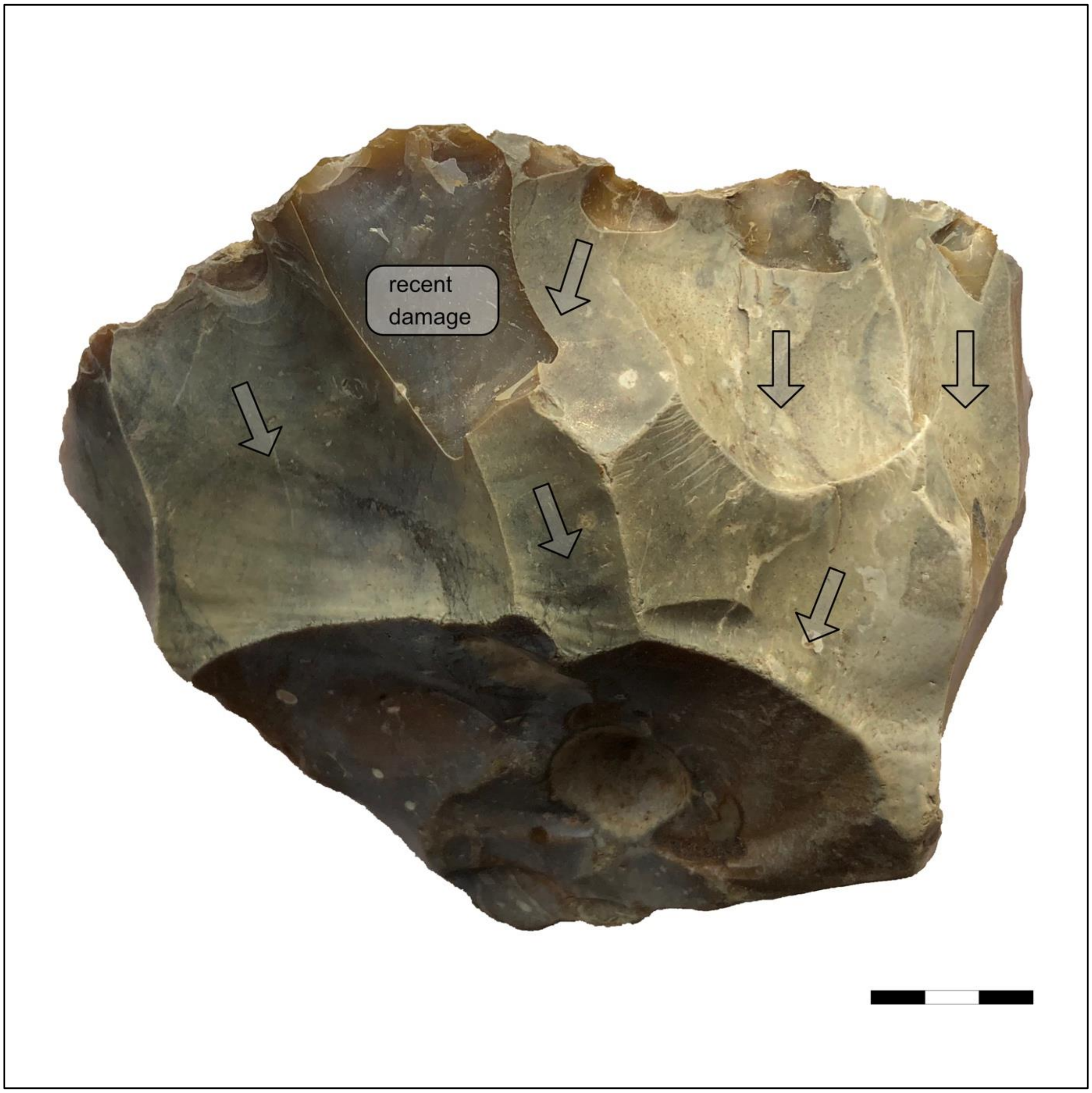

Supplementary Figure S24. Flake core (11263:1000:31) with one exploiting surface found in the Schladebach pit. Photo: M. Weiss.

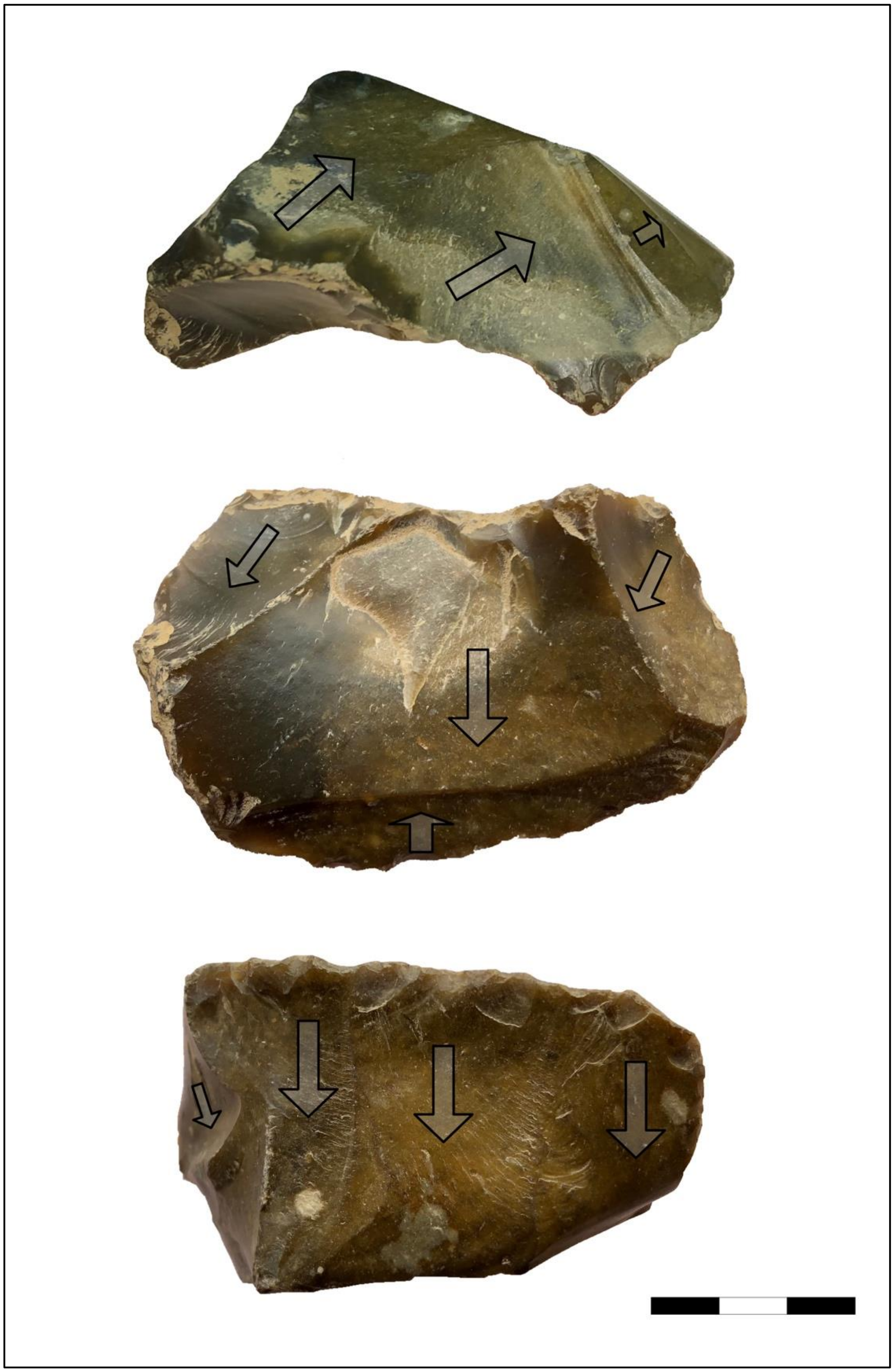

Supplementary Figure S25. Flake core (11263:1000:32) with three exploiting surfaces found in the Schladebach pit. Photo: M. Weiss.

Supplementary Table S3: Complete flakes discovered in Schladebach during survey.

The edge preservation indicates that the artefacts were reworked. A Middle Paleolithic feature, platform faceting (Platform: SCARS), was only discovered on one specimen. Different states of dorsal working show that the flakes derive from different stages within the operational chain of blank production.

INVENTORY NUMBER	EDGE PRESERVATION	LENGTH	WIDTH	THICK	PLATFORM	AMOUNT OF PLATFORM SCARS	AMOUNT OF DORSAL SCARS
11263:1000:1	HEAVILY DAMAGED	52.97000122	37.22000122	9.5	SCARS	100%	100%
11263:1000:9	LIGHTLY DAMAGED	55.75999832	62.91999817	18.87999916	CORTICAL	0%	10-30%
11263:1000:11	LIGHTLY DAMAGED	51.02999878	48.24000168	14.5	PLAIN	100%	40-60%
11263:1000:12	LIGHTLY DAMAGED	57.75	30.77000046	17.88999939	SCARS	100%	70-90%
11263:1000:13	ROLLED	75.26000214	30.72999954	20.79999924	PLAIN	100%	40-60%
11263:1000:14	HEAVILY ROLLED	39.47999954	31.14999962	14.59000015	PLAIN	0%	100%
11263:1000:15	LIGHTLY DAMAGED	30.86000061	26.19000053	11.13000011	CORTICAL	0%	70-90%
11263:1000:16	HEAVILY ROLLED	39.63999939	29.94000053	11	PLAIN	100%	100%
11263:1000:21	HEAVILY DAMAGED	42.56000137	30.89999962	8.890000343	CORTICAL	0%	70-90%
11263:1000:23	LIGHTLY DAMAGED	51.31999969	44.27999878	16.20999908	CORTICAL	0%	70-90%
11263:1000:24	ROLLED	50.88999939	37.70999908	17.30999947	CORTICAL	0%	100%
11263:1000:25	HEAVILY DAMAGED	41.70999908	33.81000137	15.85000038	PLAIN	100%	40-60%
11263:1000:28	HEAVILY ROLLED	35.72000122	36.97999954	13.64999962	PLAIN	0%	0%

Supplementary Table S4: Complete cores discovered in Schladebach during survey.

The edge preservation indicates that most of the cores were reworked. Generally, no cores of Middle Paleolithic character are present: they have mostly an irregular shape and no prepared core edges and striking platforms.

INVENTORY NUMBER	EDGE PRESERVATION	LENGTH	WIDTH	THICK	CORE SHAPE	FLAKING DIRECTIONs	NUMBER OF PREPARED CORE EDGES	CONDITION OF THE STRIKING PLATFORM
11263:1000:18	FRESH	32.77999878	53.90000153	16.64999962	IRREGULAR	BIDIRECTIONAL	0	CORTICAL
11263:1000:19	HEAVILY ROLLED	61.52000046	66.08000183	36.33000183	IRREGULAR	CONCENTRIC	0	CORTICAL
11263:1000:30	HEAVILY DAMAGED	99.62000275	92.55999756	80.73000336	TRIANGULAR	UNIDIRECTIONAL	0	PLAIN
11263:1000:31	ROLLED	135.7200012	148.8300018	67.25	NATURAL	UNIDIRECTIONAL	0	CORTICAL
11263:1000:32	LIGHTLY DAMAGED	51.13999939	73.93000031	30.54000092	IRREGULAR	BIDIRECTIONAL	0	PLAIN
11263:1000:33	HEAVILY ROLLED	53.58000183	44.81000137	32.70999908	OVAL	CONCENTRIC	0	PLAIN
11263:1000:34	HEAVILY DAMAGED	53.77000046	57.29000092	30.69000053	IRREGULAR	UNIDIRECTIONAL	0	CORTICAL
11263:1000:35	LIGHTLY DAMAGED	54.88000107	41.45999908	26.22999954	IRREGULAR	BIDIRECTIONAL	0	PLAIN

2. Rehbach

At Rehbach, the fluvial sand- and gravel of the SMT (Supplementary Fig. S26) are preserved between the 1nd Elsterian till and the Saalian till of the Zeitz phase (Drenthe stage). The 1st Elsterian till is eroded. Only a stone pavement as a till-residuum can be observed above the 1st Elsterian till. The fluvial sediments overlying the Elsterian till yield luminescence ages of 423 ± 48 and 387 ± 48 ka. These sediments were

only preserved in remnants at the base of the SMT. The main aggradation period started at around 239 ± 31 ka. The corresponding sand- and gravel are preserved throughout the entire SMT exposure and are associated with the Middle Paleolithic artefacts, as shown by a potential flake (Supplementary Fig. S27).

The fluvial sediments underlying the Saalian till are characterized by ice wedges and cryoturbation features. At the very top part of the SMT, the silt-rich sediments of the so called Markkleeberger silt- and cryoturbation horizon are preserved. These deposits are covered by the glaci-limnic sediments of the Böhlern warved clays (“Bändertone”) and the up to 2 m thick till.

The very top part of the SMT is dated to 160 ± 13 – 144 ± 13 ka at Rehbach and 159 ± 17 – 164 ± 16 ka in Markkleeberg, yielding maximum ages for the ice advance of the Zeitz phase and putting the Zeitz-phase into late MIS 6.

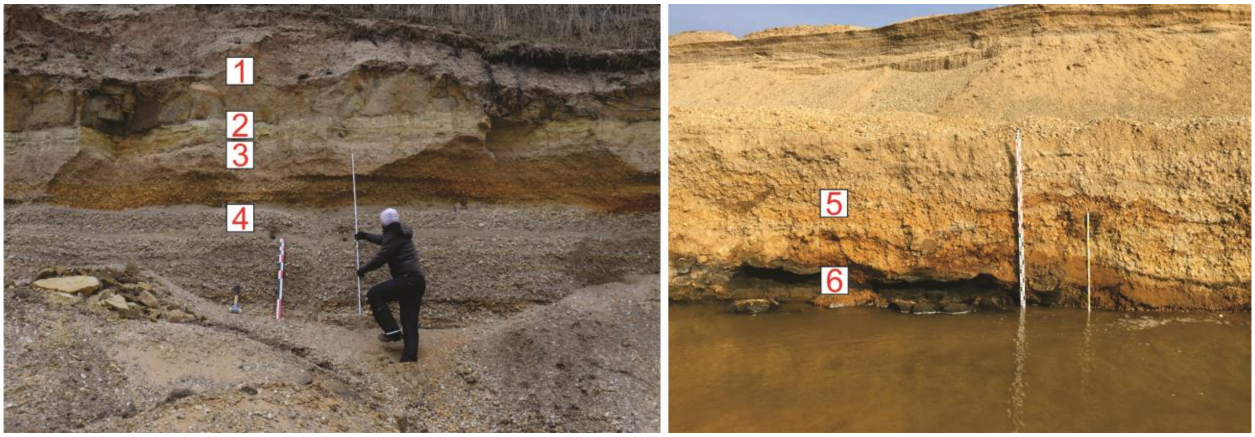

Supplementary Figure S26. Stratigraphic situation in the Rehbach pit. Photo: M. Weiss/ T. Lauer.

1 = Saalian till (Zeitz-phase); 2 = Pro-glacial warved lake deposits (“Böhlener Bänderton”); 3 = Markkleeberg cryoturbation horizon; 4 = SMT fluvial sand and gravel with ice wedges (Weiße Elster river); 5 = Basal SMT unit; 6 = till of the 1st Elsterian ice advance (the till of the 2nd ice advance is eroded).

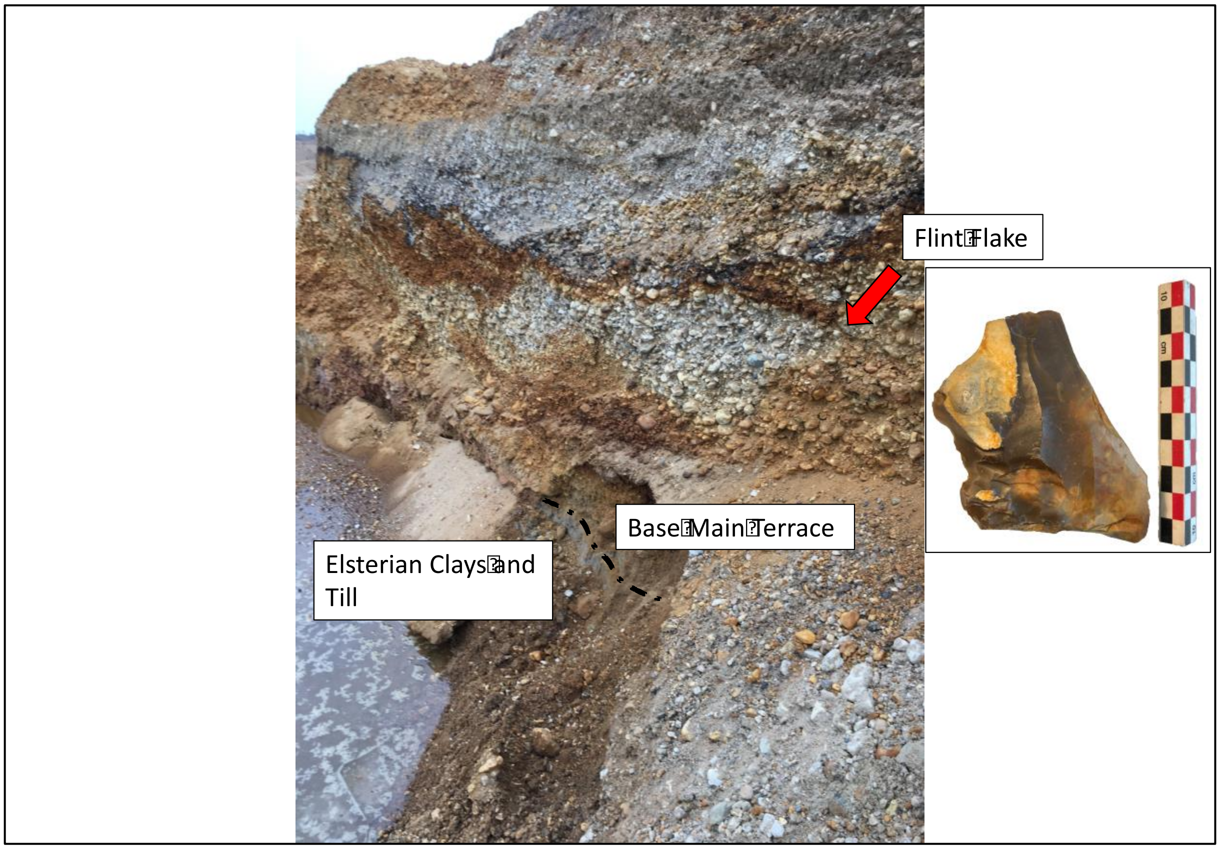

Supplementary Figure S27. Basal part of the SMT in Rehbach. A broken and damaged, potential flake (REH-09/1/1) was found within the fluvial sand- and gravel of the main aggradation period of the sequence, starting around 239 ± 31 ka. Photo M. Weiss.
